# The importance of naming cryptic species and the conservation of endemic subterranean amphipods

**DOI:** 10.1038/s41598-017-02938-z

**Published:** 2017-06-13

**Authors:** Teo Delić, Peter Trontelj, Michal Rendoš, Cene Fišer

**Affiliations:** 1 SubBio lab, Department of Biology, Biotechnical Faculty, University of Ljubljana, Večna pot 111, Ljubljana, 1000 Slovenia; 2 State Nature Conservancy, Slovak Caves Administration, Hodžova 11, 031 01 Liptovský, Mikuláš Slovakia

## Abstract

Molecular taxonomy often uncovers cryptic species, reminding us that taxonomic incompleteness is even more severe than previous thought. The importance of cryptic species for conservation is poorly understood. Although some cryptic species may be seriously threatened or otherwise important, they are rarely included in conservation programs as most of them remain undescribed. We analysed the importance of cryptic species in conservation by scrutinizing the South European cryptic complex of the subterranean amphipod *Niphargus stygius sensu lato*. Using uni- and multilocus delineation methods we show that it consists of 15 parapatric and sympatric species, which we describe using molecular diagnoses. The new species are not mere “taxonomic inflation” as they originate from several distinct branches within the genus and coexist with no evidence of lineage sharing. They are as evolutionarily distinct as average nominal species of the same genus. Ignoring these cryptic species will underestimate the number of subterranean endemics in Slovenia by 12 and in Croatia by four species, although alpha diversity of single caves remains unchanged. The new taxonomy renders national Red Lists largely obsolete, as they list mostly large-ranged species but omit critically endangered single-site endemics. Formal naming of cryptic species is critical for them to be included in conservation policies and faunal listings.

## Introduction

Invertebrates comprise the majority of metazoan diversity, yet only a small fraction of them has been included in nature conservation. Cardoso *et al*.^1^ listed no less than seven causes impeding their inclusion into contemporary conservation. The most common and the most fundamental drawback is taxonomic incompleteness, leading to incomplete knowledge of species distributions, ecology, population dynamics, but also lower public interest in those species^1^. Even though taxonomic incompleteness is an old and well-known problem in conservation, molecular taxonomy^2, 3^ has unveiled that the taxonomic impediment may be much deeper than previously thought. Several authors reported that nominal species may count tens of morphologically near-identical but genetically distinct species^4–6^, so called cryptic species^7^. These are a rather common evolutionary phenomenon, widespread across all animal phyla^8^, and they apparently contribute a substantial part to the global species richness.

Cryptic species are the worst-case scenario of taxonomic incompleteness. They are detected and delineated, but usually remain undescribed and unavailable to conservation practice^9^. Moreover, the biological properties relevant for the conservation of cryptic species are in most cases not known. Some cryptic species may be evolutionary young, in a stage of transition from population to species, where morphologies of descendants have not yet diverged^10^. Such species might be considered as less important for conservation^11^. Other cryptic species may be phylogenetically old and reproductively isolated from each other by strong biological barriers^12^. Recent studies indicate such species may be inappropriately managed^13^ and much more threatened than previously thought^14, 15^. Yet, the degree to which our failure to recognize and include cryptic species in conservation programs affects biodiversity conservation in general, remains largely unexplored. It is timely to change our attitude towards cryptic species and consider them as an important part of the real world^8^ rather than a rare phenomenon. These species deserve our full attention^16^, an appropriate taxonomic treatment including naming^17–19^ and careful study of their biological traits to appropriately assess their value for conservation^20^.

**Figure 1:**
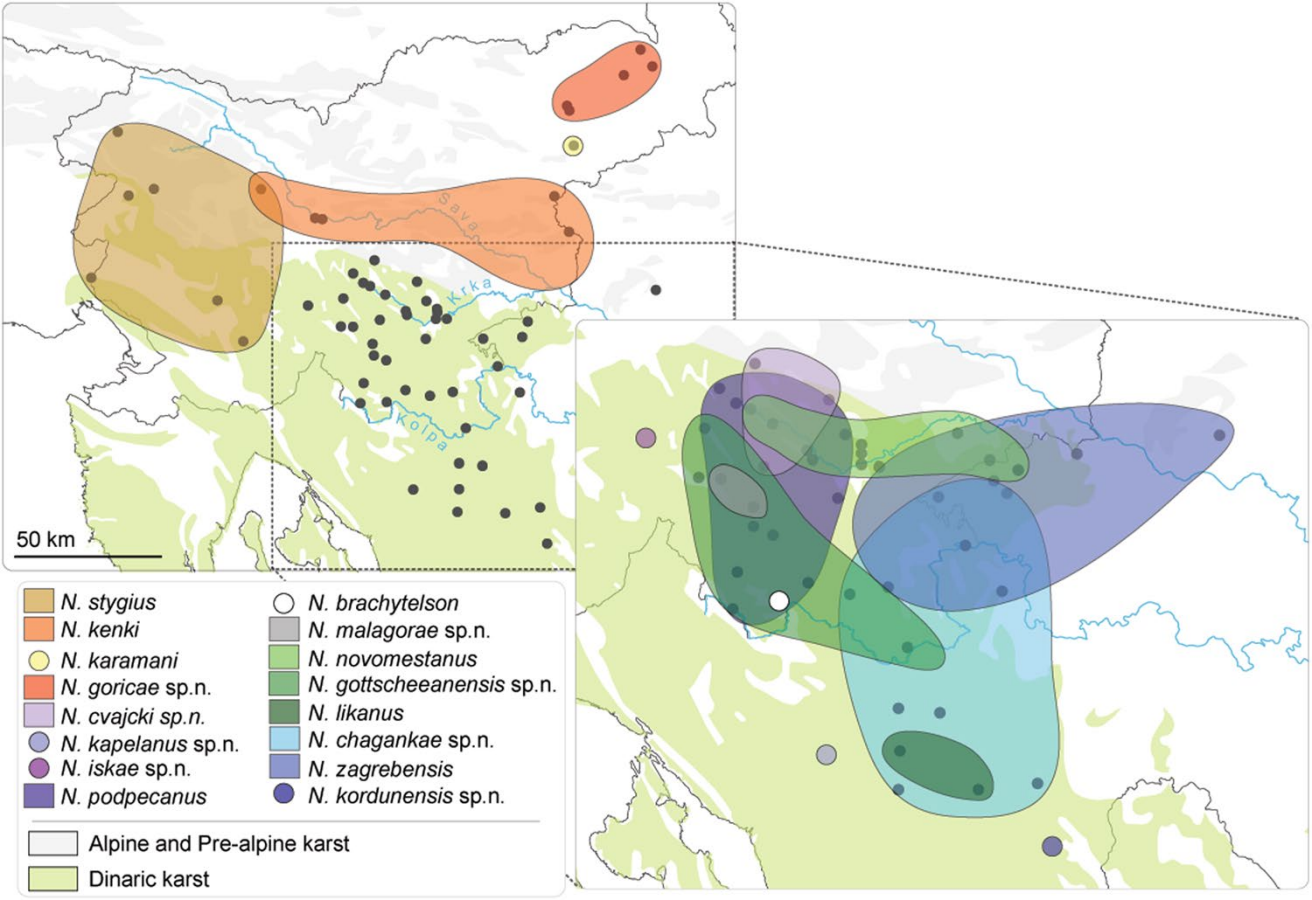
Distribution of species of the *Niphargus stygius* complex. Sampling sites are represented as dots with overlaid approximate ranges based on known sites. Sampling localities were GPS georeferenced and a basic layout was produced in ArcGis 10.1 (http://www.esri.com/software/arcgis/arcgis-for-desktop), the exported image was then edited in Adobe Illustrator CS6 (http://www.adobe.com/support/downloads/product.jsp?platform=Windows&product=27).

Here we present a case study on subterranean amphipod crustaceans from Southern Europe. We disentangle the taxonomy of the complex, and apply various conservation metrics that consistently put cryptic species high on conservation priority lists. The studied group is a complex of morphologically similar populations of *Niphargus stygius* (Schiödte, 1847) distributed in Slovenia and North-Western Croatia (Fig. 1). Stanko Karaman attempted to split the complex into seven subspecies^21^. The proposed diagnostic traits, however, turned out to be too unreliable for identification^21^. When additional morphological studies (Supplementary Material 3) failed to provide diagnostic characters to distinguish between members of the complex, it became clear that the taxonomy of the most common amphipods in caves and springs in the region cannot be resolved on a morphological basis. Preliminary molecular study revealed a high genetic lineage diversity^22^ and prompted for a revision of the complex.

Our study has three aims. We first test the conjecture that the complex comprises multiple cryptic species, and apply uni- and multilocus means to identify and delimitate them. Second, we explore whether and how considering these cryptic species in faunal listings affects conservation priorities. Specifically, we studied how changes in taxonomy affect regional species richness, range size and evolutionary distinctness. Third, we describe the species. Based on our results we conclude that naming cryptic species does matter as it can strengthen and optimize conservation decisions.

## Results

### Phylogenetic analyses and species delimitation

**Figure 2:**
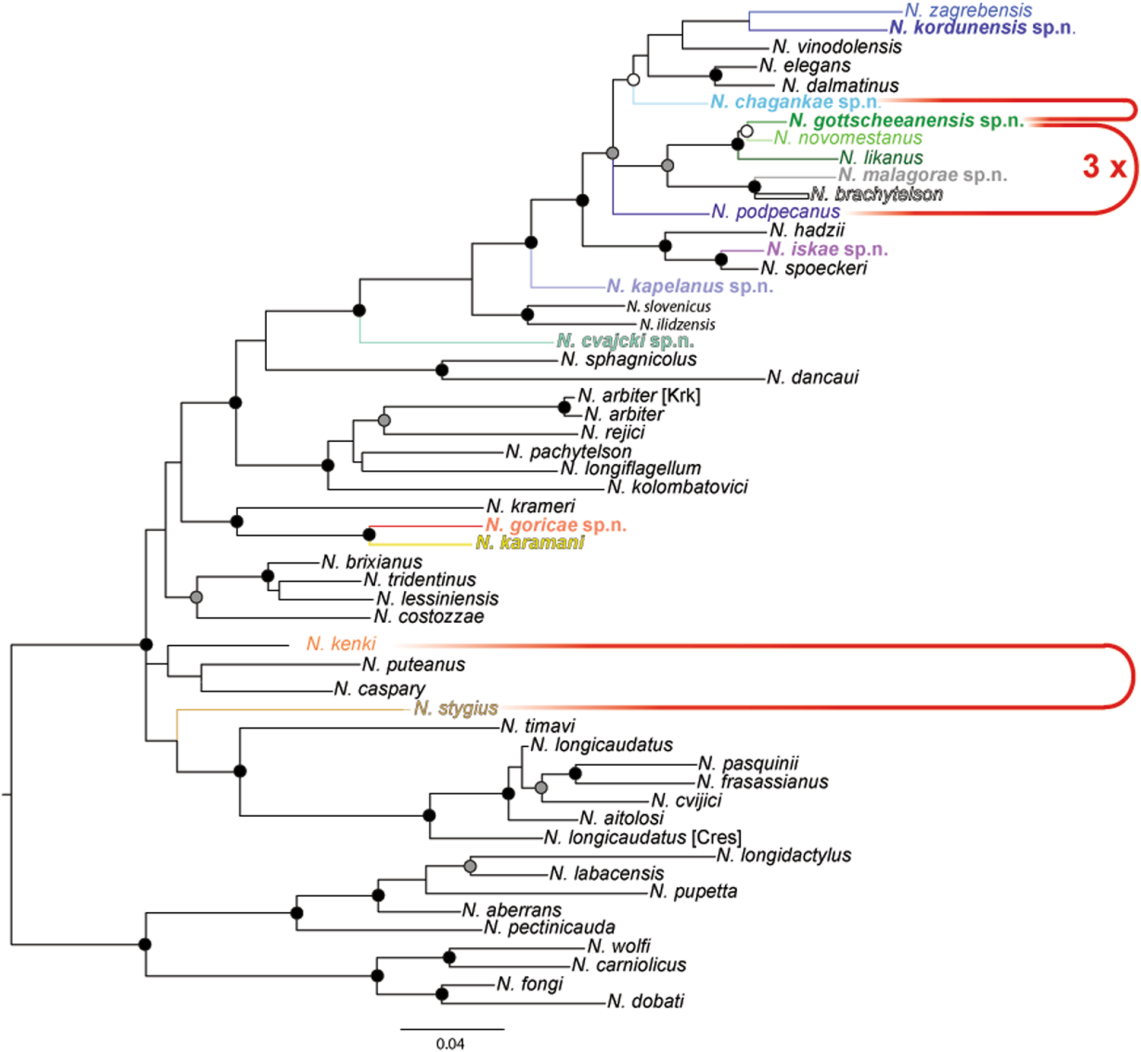
Phylogenetic tree reconstructed using Bayesian inference. Black and grey dots indicate posterior probabilities of 1 and >0.95, respectively. Species of the *Niphargus stygius* complex are marked with colours as per Fig. 1. To simplify the presentation, we collapsed terminals of each species. The full species delimitation tree with 164 individuals is available in Supplementary Information 2. Species found in syntopies are labelled with red dots. Altogether, five syntopies were recorded; of these *N. podpecanus* and *N. gottscheaensis* were found three times together.

We analysed 104 individuals from the studied complex collected at 64 localities (Fig. 1). In order to explore the phylogenetic position of the focal taxa, we compiled a dataset with additional 37 species (details in Supplementary Information, Table S1) covering all main *Niphargus* lineages^22, 23^. Bayesian and Maximum Likelihood analyses yielded identical tree topologies (Fig. 2, Fig. S1). The studied group of taxa was not monophyletic but split into four major non-sister lineages. The first three lineages represent the nominal *N. stygius stygius* from West Slovenia, the nominal *N. s. karamani* from North-Eastern Slovenia and the nominal *N. s. kenki* distributed between Central and Eastern Slovenia along the Sava River. All remaining species are nested within a strongly supported clade together with already described and morphologically distinct species *N. illidzensis, N. slovenicus, N. dalmatinus, N. vinodolensis. N. zagrebensis* and *N. elegans* (Fig. 2, Fig. S1). This last clade is distributed between the lowlands of northern Italy, the northern Dinaric Karst and the western margins of the Pannonian Plain.

Species hypotheses were inferred using uni- and multi-locus tree-based^24, 25^ species delimitation methods, and unilocus distance-based^26^ methods. We first ran the unilocus Poisson Tree Processes (PTP)^24^ delimitation procedure. The analyses were performed on a COI alignment counting 117 sequences belonging to herein studied and 37 other species (Supplementary Information 1, Table S1). The results suggest that the studied species complex counts 16 distinct species. Nominal *N. s. brachytelson, N. s. novomestanus, N. s. kenki* and *N. s. karamani* each split into two distinct species, nominal *N. s. podpecanus* into three, and *N. s. likanus* into four species. Only nominal *N. s. stygius* remained a well-supported single species. In addition to the focal species complex, the analyses unveiled another nominal taxon hiding additional cryptic diversity, i.e., the nominal *N. zagrebensis* turned out to consist of two morphologically similar species. Next, we applied a multilocus coalescence delimitation method using Bayesian Phylogenetics & Phylogeography 3.1 (BPP)^25^. As the *N. stygius* s. lat. species complex turned out to be non-monophyletic, we ran this analysis separately for each of three lineages that tentatively included additional species. Multilocus species delimitation confirmed the results of the unilocus analysis, regardless of the population size and root age priors used (Fig. 2, Table S3).

**Figure 3:**
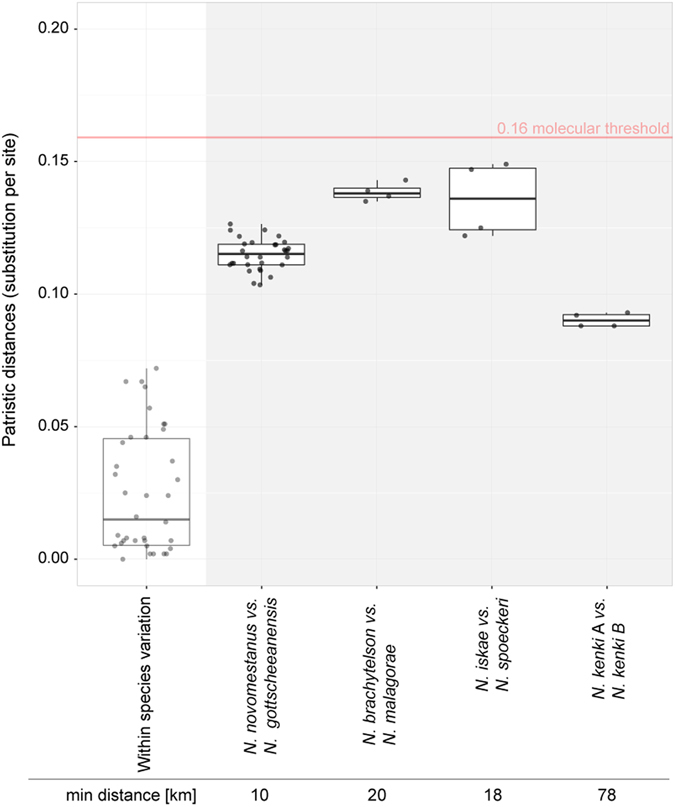
Patristic genetic distances based on COI gene sequences. Only those putative species are shown that diverged less than the proposed threshold of 16%. Within-species distances, pooled for all eight putative species (left boxplot), are substantially lower than between-species distances calculated for all four pairs of species (right boxplots). Three pairs are geographically close (nearest distances among sampling sites indicated at the bottom), whereas the least divergent pair of hypothetical species is geographically distant.

In order to assure robustness of species hypotheses, we supplemented the tree-based species delimitations by a distance-based approach. We tested whether COI sequences of the putative species diverged more than 16% in patristic and 4% in Kimura-Two-Parametric (K2P) distances. The two thresholds are conservative estimates of lower species boundaries, determined empirically. The 16% patristic distance corresponds to the divergence among 276 well-defined crustacean species^26^, whereas a 4% K2P distance indicates the divergence beyond which interbreeding among *Gammarus* amphipods becomes unlikely^27^. Pairwise comparisons of the delimited species indicate high molecular divergence between most species pairs. All species pairs exceeded the 4% K2P threshold, and all but four species pairs exceeded the 16% patristic distance threshold (Table S4, Fig. 3). However, even in these four species pairs, interspecific distances substantially exceed intraspecific ones (Fig. 3).

As a final criterion, we used sympatric occurrence of cryptic phyletic lineages as a pointer to reproductive isolation and hence distinct species even under the biological species concept (Figs 1 and 2). We found that out of the 16 delimited species ten pairs had partially overlapping ranges (Fig. 1). In five caves, we encountered species pairs in syntopy (i.e., three species pairs, of these one pair syntopically co-occurred three times). These observations we treated as natural crossing experiments, and they revealed no sign of gene flow or introgression between species. Among one mitochondrial and three nuclear loci all haplotype were consistently species-specific.

Four boundary cases, which diverged less than 16% in their patristic COI distances, are allopatric. However, a close examination unveils that in three of these species pairs divergences arose over short geographic distances (10–20 km, Fig. 3). These species pairs were found within the same catchments, indicating that the observed divergences and uniqueness of lineages may be explained by biological reproductive barriers rather than limited dispersal. Less clear is the case of the least diverged species pair (*N. kenki* complex; 9% patristic and 5.5% of K2P distance) separated by 80 km. Although all delimitation methods but one suggest this last pair of populations should be treated as separate species, the data are insufficient to rule out alternative explanations, such as isolation by distance. For this reason we decided to postpone taxonomic decisions about distinct *N. kenki* populations until additional data clarify their status.

### Impact of cryptic species on species richness

The results of the species delimitation procedures substantially affected species richness measures. The species number has risen by a factor of two and up to a factor of 15, depending on whether traditional subspecies are treated as species^28^ or not^21, 29^. We analysed the effects of change in species richness at two geographic scales that are relevant for conservation: at single sites and nation-wide. As the complex is spread across the Danube and Adriatic basins, we investigated whether species richness equally increased in the two basins.

At the level of individual caves, the effect of increased species richness was negligible. Species richness has increased by one species in five caves where syntopic occurrences of two species were detected. On a nation-wide scale, the number of species increased by a factor of 12 in Slovenia and by a factor of five in Croatia. Interestingly, if subspecies were treated as phylogenetic species^28^, the increase in species richness in both countries would be approximately two fold (1.9 for Slovenia and 1.7 for Croatia). Increase in species richness in the two basins was strongly asymmetric. All newly discovered cryptic species were detected in the Danube basin.

### Changes in range size – a proxy of species vulnerability

Changes in range sizes were remarkable. The range of the nominal *N. stygius sensu* S. Karaman, including all its subspecies, measured over 20,000 km^2^, spanning East Italy, Slovenia and West Croatia. This range is unusually large for an aquatic species living in subterranean environment^12^. It is further unusual in that it covers the Adriatic and the Black Sea Drainage, as well as four major European biogeographic regions: the Alpine, the Mediterranean, the Dinaric and the Pannonian^30^. By contrast, ranges of cryptic species within the complex are much narrower. Five species are known from a single locality only and two species are known from two localities. Among species known from more than three localities, three have ranges smaller than 500 km^2^, and six have ranges between 500 and 5000 km^2^. As for comparison, according to the IUCN Red List criteria, range sizes below 5000 km^2^ indicate species are endangered^31^.

### Evolutionary distinctness of cryptic species

Another measure relevant for conservation is evolutionary distinctness (ED), a metrics that estimates evolutionary uniqueness of a species as compared to its congeners^32, 33^. The measure may be related also to a species’ function in the ecosystem (see Discussion)^34^. ED is a measure of a species’ terminal branch-length, corrected for the species richness of the containing clade; i.e., species on longer branches with fewer congeners receives higher ED value than species within recent, species rich radiations^32^. It is measured either in millions of years or in the amount of nucleotide substitutions accumulated along the branch of a phylogenetic tree.

**Figure 4:**
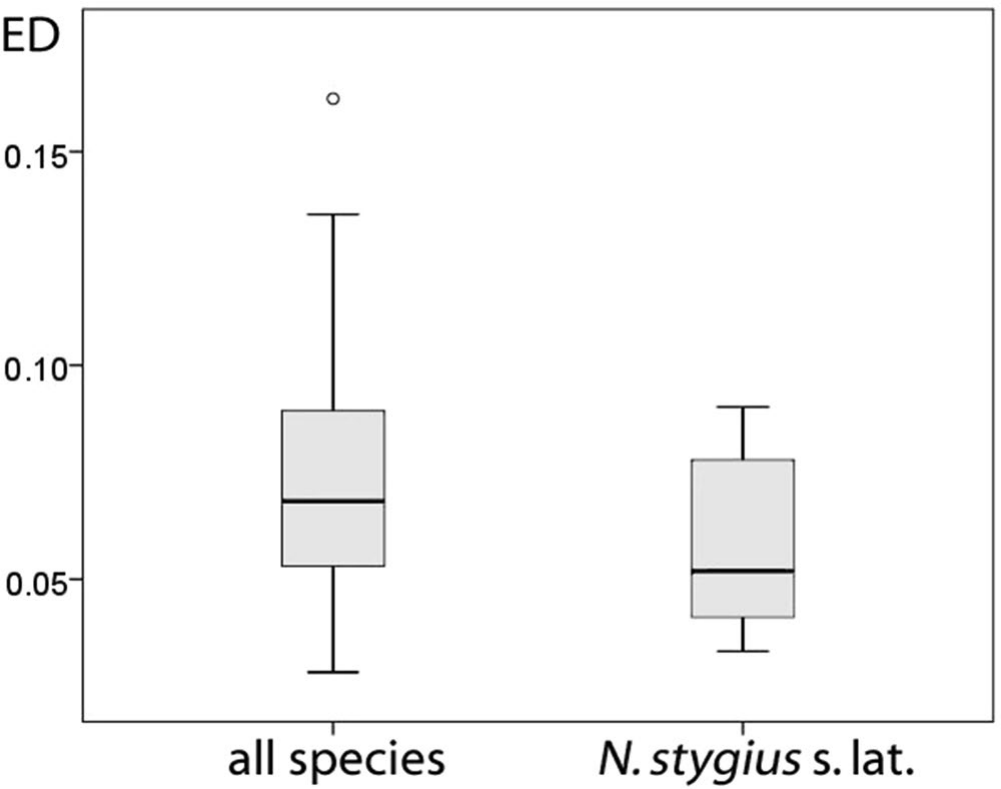
Distribution of evolutionary distinctness (ED) of the studied *Niphargus stygius* sensu lato compared to the distribution of evolutionary distinctness of other analysed species. Evolutionary distinctness of the studied complex is slightly, but not significantly lower (Mann-Whitney U test, P = 0.092).

The evolutionary distinctness of *N. stygius* as traditionally conceived was 0.087 nucleotide substitutions per site. As cryptic species belonged to four independent clades within the wider phylogeny of *Niphargus*, their ED is not diminished by splitting. The ED values of the herein studied species of the *N. stygius* complex differed from each other by a factor of 2.4 (0.037–0.087, Table 1). As these values are little informative *per* *se*, we compared them to the values calculated for the other, non-cryptic species included in our phylogenetic analysis. About one third of the species attained relatively high (>0.075), and another third low (<0.045) ED values (Fig. 4). Overall, the ED values of the studied complex were only slightly, but not significantly lower than in the non-cryptic species from the rest of the phylogeny (Mann-Whitney U test, P = 0.092).

**Table 1:** Range sizes, evolutionary distinctness (ED), and estimated conservation importance of members of the *N. stygius* species complex.

species	Range size surface [km^2^]/maximum range diameter [km]	ED [substitutions per nucleotide site]	Conservation importance^1^
*Niphargus brachytelson*	Single site/0	0.052	2b
*Niphargus chagankae* sp. n.	1679/50	0.042	3
*Niphargus cvajcki* sp. n.	139/20	0.080	2a
*Niphargus goricae* sp. n.	142/32	0.078	2a
*Niphargus gottscheeanensis* sp. n.	1946/65	0.045	3
*Niphargus iskae* sp.n.	Single site/0	0.049	2b
*Niphargus kapelanus* sp. n.	Single site/0	0.037	2b
*Niphargus karamani*	Single site/0	0.074	1
*Niphargus kenki*	1001/106	0.056	2b
*Niphargus kordunenensis* sp. n.	Single site/0	0.084	1
*Niphargus likanus*	Two sites/37	0.055	3
*Niphargus malagorae* sp. n.	Two sites/9	0.064	3
*Niphargus novomestanus*	228/29	0.038	3
*Niphargus podpecanus*	871/46	0.051	3
*Niphargus stygius*	2922/85	0.087	2a
*Niphargus zagrebensis*	1193/80	0.090	2a

Range sizes are given as km2 or as maximal linear distance of the range to make data compliant with IUCN Red List^31^ and the rest of publications from subterranean biology, respectively.

^1^Conservation ranks are defined as follows: (1) high endemism and ED; (2a) low endemism, high ED, (2b) high endemism, low ED; (3) low endemism and ED.

### Species diagnoses and names

We showed that, according to different criteria, the 15 delimited genetic lineages represent fully evolved species that likely meet even the most rigorous criteria of biological species^35^. Moreover, because of their small ranges, exceptionally high endemism, and their evolutionary distinctness, they are highly relevant for conservation. We therefore undertook the necessary formal steps to name and diagnose them as species under the provisions of The International Code of Zoological Nomenclature (The Code). We raise seven existing subspecies to species rank, and diagnose nine species new to science.

The morphological diagnosis for the entire complex as provided by S. Karaman^21^ remains valid and is available in Supplementary Information 3. For diagnosing the species, we followed recent recommendations^17, 18^ (details in Material and Methods); the analyses were made in CAOS^36^. All species were diagnosed using unique combination of character attributes at four genes. The number of diagnostic characters varied between 0 and 156 per marker (Table 2). Specimens from localities from which subspecies were described were considered as representatives of already named taxa. An intriguing problem was to assign the name *N. podpecanus* to the right species. In its type locality, the cave Podpeška jama, we found two co-occurring, genetically distinct but morphologically indistinguishable species. The type series is approximately 70 years old and no longer contains any useful DNA. However, in his original work, S. Karaman^21^ reported that the species was found also in the vicinity of the city of Kočevje. In order to maximize compatibility between the bibliographic record and our new taxonomic conclusions, we assigned the name *N. podpecanus* to the one species that we found also in Kočevje. Following Article 75 of the Code, we erected a neotype for *N. podpecanus* (Table 2).

**Table 2:** Molecular diagnoses of species within the *Niphargus stygius* complex, including species redescriptions and newly described species.

Species (ZooBank lsid/WoRMS lsid)	Type locality	Holotype specimen/reference specimen – collection voucher numbers	Molecular diagnosis**
*Niphargus chagankae* sp. n. (4DF5BD7A-B343-4354-AC65-F7FFFA829123/988191)	Cave Čaganka, Poljanska gora, Črnomelj, Slovenia	NB792	**28 S**:- **COI**: 228 G, 262 T, 264 A, 300 A **ITS**: 98 G,540 C,877 A,1027 T,1050 G,1084 C,1152 A,1172 G,1178 G,1200 -,1201 -, 1202 -,1204 -,1209 -,1211 -,1212 -,1213 -,1217 -,1218 -,1221 -,1222 -,1223 -,1229-, 1233 -,1235 -,1238 -,1241 -,1638 T,1682 T,1726 C,1727 T,1728 C,1737 T,1741 T,1898 C,2055 T,2065 A,2070 -
*Niphargus cvajcki* sp. n. (A6B6A0BC-20BC-4109-BAAA-72588484BC5A/988197)	Cave Šolnovo brezno, Prevole, Žužemberk, Slovenia	NB915	**28 S:** 74 G, 78 C, 81 T, 136 -,160 A, 196 T, 203 G, 209 A **COI**: 3 T, 300 G, 303 G, 360 T, 411 T, 418 A, 419 G, 537 G **ITS:** 62 A, 94 T, 124 C, 209 G, 462 T, 613 C, 707 C, 709 -, 712 A, 715 C, 794 C, 809 G, 835 C, 836 G, 840 C, 846 A, 868 G, 873 A, 881 T, 888 G, 897 C, 902 C, 906 A, 1013 G, 1015 C, 1032, 1038 A, 1044 T, 1054 C, 1055 A, 1056 G, 1062 T, 1162 T, 1166 G, 1171 A, 1172 T, 1220 G, 1242 T, 1250 G, 1251 C, 1253 T, 1260 G, 1261 G, 1273 G, 1278 A, 1288 C, 1293 G, 1301 C, 1305 C, 1307 T, 1310 A, 1314 G, 1315 T, 1316 A, 1318 T, 1327 G, 1331 A, 1333 C, 1334 C, 1335 G, 1336 C, 13338 T, 1339 C, 1340 T, 1343 G, 1345 G, 1346 T, 1353 A, 1354 C, 1355 T, 1360 G, 1362 G, 1364 A, 1367 T, 1369 C, 1371 A, 1377 A, 1380 T, 1382 G, 1384 A, 1385 A, 1387 A, 1388 C, 1392 A, 1394 C, 1395 C, 1397 C, 1398 T, 1399 T, 1401 A, 1402 A, 1409 G, 1428 A, 1431 T, 1434 A, 1440 T, 1443 G, 1446 T, 1447 A, 1454 C, 1455 A, 1472 G, 1479 C, 1481 T, 1497 G, 1499 C, 1533 T, 1546 G, 1547 T, 1551 G, 1552 T, 1554 G, 1557 G, 1563 A, 1564 T, 1567 A, 1589 G, 1590 G, 1595 T, 1600 G, 1612 G, 1644 G, 1652 T, 1655 G, 1668 G, 1673 A, 1680 G, 1688 G, 1690 T, 1697 C, 1702 C, 1706 A, 1813 G, 1832 A, 1835 T, 1838 A, 1844 T, 1849 T, 1850 A, 1857 C, 1858 A, 1859 G, 1864 A, 1865 T, 1879 G, 1888 T, 1894 C, 2032 C, 2033 T, 2034 G, 2037 T, 2045 A, 2046 G, 2048 T, 2080 A, 2099 T
*Niphargus goricae* sp. n. (A0A0651F-685D-45D4-9477-5E44408D4CF3/988195)	Water well by the house Fram 119, Fram, Maribor, Slovenia	NA085	**28 S**:113 A, 115 C, 147 T, 148 C, 149 C, 168 A, 176 T, 179 A, 182 A, 185 T, 200 A, 203 T, 205 C, 207 G, 208 A, 210 G, 269 G, 312 T, 341 C, 344 G, 346 T, 362 T, 451 G, 456 A, 460 T, 491 C, 532 T, 535 A, 573 C, 722 C **COI:**204 C, 375 C, 402 C, 408 G, 441 C, 456 G **ITS**: 159 A, 161 C, 162 A, 165 G, 186 T, 213 A, 420 -, 444 G, 445 C, 504 C, 506 G, 519 G, 590 -, 605 C, 619 G, 706 C, 707 T, 708 A, 710 C, 711 A, 717 A, 737 A, 829 A, 839 G, 842 T, 843 C, 851 A, 856 T, 857 A, 1064 G, 1066 T, 1068 T, 1071 T, 1086 T, 1102 C, 1131 G, 1156 -, 1189 T, 1232 C, 1245 T, 1610 T, 1611 T, 1614 A, 1617 C, 2061 A, 2062 C, 2066 T, 2079 G, 2081 T, 2090 C, 2099 C, 2101 G, 2102 G, 2104 A
*Niphargus gottscheeanensis* sp. n. (1EEEF2DC-8016-40CE-AB83-3E142905EAB2/988192)	Cave Željnske jame, Željne, Kočevje, Slovenia	NB488	**28 S**:182 T, 212 A **COI:** 79 G, 381 G **ITS:** 1040 G, 1041 T, 1175 C, 2005 A
*Niphargus iskae* sp. n. (840AD88E-59E9-4969-ABBE-C4EFEB8D02D9/988194)	Spring on the foothill of Mačji rep, Škrabče, Nova vas, Slovenia	NC087	**28 S**:na **COI:**138 C, 318 T, 360 C, 402 A, 466 G, 470 C, 482 C, 491 G **ITS:** na
*Niphargus kapelanus* sp. n.v (10A49E2A-C93C-4114-A6EF-54374FE89AE1/988196)	Cave špilja pod Mačkovom dragom, Bjelolasica, Ogulin, Croatia	NB625	**28 S**:770 G **COI:**75 G, 216 A, 342 A, 345 C, 355 A, 435 A **ITS**: na
*Niphargus kordunensis* sp. n. (2A2EBA04-D1C8-4024-A1B0-D9BBE0E93CFD/988190)	Matešička špilja, Matešići, Slunj, Croatia	NB623	**28 S**:128 C, 131 A, 142 T, 145 T, 146 C, 153 C, 156 A, 220 A, 249 C, 676 T, 769 A **COI:** na **ITS:** na
*Niphargus malagorae* sp. n. (333C1C2D-DF19-4B13-826D-C57782EF2864/988193)	Cave Mivčje jama, Gornje Lepovčje, Ribnica, Slovenia	NB858	**28 S**:472 A **COI**: 237 G, 432 C, 495 C, 555 C **ITS**: 75 T, 77 G, 79 T, 82 A, 127 C, 582 T, 587 A, 624 -, 625 -, 632 C, 635 G, 643 A, 851 G, 881 A, 885 G, 886 T, 887 A, 943 T, 1040 C, 1075 G, 1076 T, 1078 T, 1089 C, 1112 G, 1117 G, 1122 C, 1125 -, 1126 T, 1129 A, 1133 T, 1135 G, 1137 G, 1214 G, 1224 T, 1225 A, 1248 C, 1261 T, 1307 G, 1335 A, 1347 T, 1436 C, 1457 A, 1460 G, 1461 A, 1462 G, 1466 G, 1474 T, 1476 A, 1501 A, 1506 C, 1508 A, 1509 G, 1510 C, 1512 C, 1514 A, 1515 G, 1517 C, 1522 A, 1527 G, 1616 T, 1617 A, 1637 A, 1652 A, 1743 T, 1750 A, 1790 T, 1840 C, 1878 T, 1881 T, 1883 A, 1900 A, 1902 C, 1951 T, 2014 T, 2015 A, 2035 A, 2053 A, 2098 C
*Niphargus brachytelson* S. Karaman 1952	Lukova jama pri Zdihovem, Zdihovo, Morava, Slovenia	NA071*	**28 S**:471 C, 472 T **COI:** 345 A, 351 C, 481 C, 495 T **ITS:** na
*Niphargus hadzii* Rejic 1956	Springs of Ljubljanica river, Slovenia	NA082*	**28 S**:181 C **COI:**297 G, 491 C **ITS:** na
*Niphargus karamani* Schellenberg 1935	well near Miljana, on a riverbank of Sotla, Podčetrtek, Slovenia	NB933*	**28 S**: na **COI:**108 G, 390 A, 529 G **ITS:** na
*Niphargus kenki* S. Karaman 1952	well near Miljana, on a riverbank of Sotla, Podčetrtek, Slovenia	NA087*	**28 S**: 815 T, 819 A **COI**: 482 A, 552 C **ITS**: 1140 C, 1141 T, 1145 -, 1181 T
*Niphargus likanus* Karaman 1952	Cave system Đula – Medvednica, Ogulin, Ogulin, Croatia	NB917*	**28 S**:107 G, 112 C, 170 A, 183 A, 238 T, 516 T, 517 A, 520 T, 521 G, 522 C, 523 A, 524 A, 525 A, 526 A, 527 A, 530 G, 819 T **COI**: 276 C, 333 C, 438 C **ITS:** 96 T, 254 T, 339 G, 357 T, 371 T, 509 -, 512 T, 513 T, 794 A, 832 T, 887 T, 1097 A, 1116 G, 1656 T, 1915 C, 1918 C, 1924 G, 1926 T, 2291 T, 2348 G, 2376 A
*Niphargus novomestanus* S. Karaman 1952	Spring in Prečna, Prečna, Novo mesto, Slovenia	NA131*	**28 S**:198 T, 469 A **COI:** 165 G **ITS:** 152 G, 229 A, 486 A, 962 T, 1023 A, 1289 T, 1419 A, 1684 T, 1762 T, 1823 A, 2064 T
*Niphargus podpecanus* S. Karaman 1952	Cave Podpeška jama, Podpeč, Videm, Slovenia	NA101*	**28 S**:172 C, 387 T, 710 T, 731 A, 766 C **COI:** 333 G, 423 A, 483 A **ITS**: 775 T, 842 A, 849 A, 947 C, 989 C, 1000 A,1147 A, 1149 T, 1177 G, 1179 A, 1197 T, 1247 G, 1337 A, 1391 A, 1434 T, 1458 C,1500 C, 1512 A, 1519 G, 1522 T, 1540 A, 1542 T, 1549 C, 1569 G, 1570 A, 1589 T, 1648 A, 1733 A, 1789 C, 1800 A, 1838 T, 1899 A, 1945 A, 1957 G, 1959 C, 1988 T, 1998 T
*Niphargus spoeckeri* Schelleneberg 1933	Cave Črna jama, Veliki otok, Postojna, Slovenia	NA108*	**28 S**: na **COI:**111 G, 198 G, 318 G, 453 G **ITS:** na
*Niphargus zagrebensis* S. Karaman 1950	Vicinity of Zagreb, Croatia	NA117*	**28 S**: na **COI:** 66 G, 123 G **ITS**: 126 C, 132 T, 798 C, 828 A, 830 A, 831 C, 842 G, 882 T, 897 A, 909 T, 910 G, 924 T, 935 C, 936 A, 945 C, 948 A, 949 C, 952 G, 954 C, 958 A, 959 A, 961 A, 989 G, 1011 A, 1012 T, 1014 A, 1015 T, 1018 G, 1024 C, 1030 G, 1092 C, 1095 G, 1137 T, 1149 A, 1152 G, 1230 C, 1239 G, 1282 T, 1287 G, 1289 A, 1290 C, 1295 T, 1296 G, 1297 C, 1299 G, 1301 A, 1309 G, 1314 T, 1315 A, 1322 A, 1326 A, 1337 C, 1410 A, 1416 C, 1417 G, 1420 C, 1423 A, 1470 C, 1471 A, 1491 T, 1511 A, 1542 A, 1544 A, 1562 G, 1597 C, 1599 T, 1634 G, 1635 A, 1636 A, 1638 C, 1677 T, 1682 G, 1684 A, 1685 C, 1691 T, 1692 G, 1693 C, 1695 G, 1697 A, 1705 G, 1708 T, 1709 G, 1710 T, 1711 A, 1716 A, 1718 A, 1722 A, 1725 G, 1733 C, 1744 G, 1797 G, 1798 C, 1814 A, 1820 C, 1821 G, 1824 C, 1827 A, 1872 C, 1875 T, 1876 G, 1877 C, 1878 A, 1898 T, 1918 A, 1929 T, 1949 A, 1950 A, 1995 C, 1997 T, 2009 A, 2010 G, 2013 G, 2015 C, 2017 G, 2018 C, 2019 A, 2047 A, 2049 A, 2074 A, 2100 C

Voucher numbers refer either to specimens selected as reference specimens for previously described species or specimens selected as holotypes of newly described species. All specimens were deposited in the Zoological collection of the Department of Zoology.

^*^Asterisk denotes reference specimens of species that were described before this study. ** - denotes gap in alignment.

Species names, etymology, voucher numbers and information about type repositories are available in Table 2 and in Supplementary Information 3. The specimens and samples are available for further exploration; alignments and used for diagnosing the species are deposited at Dryad (doi:10.5061/dryad.6rs6q). In order to make descriptions compliant with The International Code of Zoological Nomenclature, the species were registered in Zoobank and World Register of Marine Species (Table 2, Supplementary Information 3).

## Discussion

The studied complex of morphologically very similar or indistinguishable species is a polyphyletic assemblage belonging to four different *Niphargus* clades. Our results indicate that the new species are not merely subspecies glorified by the use of the phylogenetic species concept. Their high conservation value, contributed mainly through endemism, but also ED, demonstrates that describing new cryptic species is necessary to bridge the deep gap between the mass production of molecular trees and the end users of taxonomic knowledge^9^. Our results further demonstrate that the recent depreciation of the taxonomic practice of raising subspecies to species, calling it “taxonomic inflation”^11, 37, 38^, is misguided for invertebrates. This conclusion strongly applies to taxa where cryptic species are overrepresented^7, 8, 39^, such as freshwater and marine amphipods with tens of morphologically cryptic species hiding behind a single nominal species^4, 40–42^. Furthermore, taxonomic inflation is unlikely to be an issue in ecological settings that produce convergent evolution through strong directional selection^39^, for example the subterranean environment^12, 43^.

Paradoxically, the effect of cryptic species on numerical selection criteria for sites important for conservation is likely to be negligible. The reason is that cryptic species rarely co-occur in syntopy, in our case only in five out of 64 localities (Fig. 1). Consequently, alpha diversity of single sites remains constant, even when regional and global species richness increases considerably. A reduction in range sizes is the expected and logical outcome of splitting a widespread morphologically defined species into a series of morphologically cryptic species^12, 44^. If *N. stygius* as conceived by S. Karaman^21^ remained the sole accepted species, it would probably be among the least endangered subterranean amphipods in Europe^12^ and as such would escape the required conservation attention. At this point it should be noted that *N. stygius stygius* as a subspecies is in fact listed on the Red List of Slovenian Malacostraca as “rare”^45^. However, in the light of the new data this listing is obviously erroneous, as it includes the species with the largest range in the complex but excludes more exposed and rarer species, even single-site endemics (Fig. 1). The Croatian Red List appropriately reflects the rareness of *N. kenki* and *N. likanus*, but fails at several other even rarer species^46^. These are clear examples of how naming of cryptic species can augment and even refute officially accepted conservation priorities.

Besides the above generalizations, our results allow us to address two issues important for the conservation of groundwater fauna in the northern Dinaric Karst, one of global hotspots for subterranean fauna^47, 48^. First, the rich and polyphyletic assemblage of new cryptic species may harbour considerable functional diversity as well. Amphipods as an important group of macroinvertebrates in groundwater^47^, are critically involved in the maintenance of groundwater ecosystem functioning^49^. The protection of evolutionary history, besides its intrinsic value, may help preserve a diversity of features important for ecosystem functioning^32–34, 50^. Even closely related cryptic species have different ecological requirements to abiotic factors^51, 52^, sensitivity to toxic chemicals or parasites (i.e. Grinnelian niche)^53, 54^, and play different roles in the ecosystem (i.e., Eltoninan niche)^55^. Narrow endemics, in particular single site endemics, are vulnerable to extinction and the loss of functional diversity by extinction of single-site endemics might have unpredictable consequences for ecosystem function at a local scale^56^. Even if replaced with another species from the complex, it may take some time to stabilize ecological processes, the worst consequence being deterioration of groundwater quality.

Second, asymmetric speciation, which took place in the Danube basin but not in the Adriatic basin, opens a new perspective on the protection of freshwater fauna in the region. In Slovenia, the Adriatic drainage basin represents 19% of the total surface, but harbours about one half of all fish species on the Slovenian Red List, including several narrowly endemic species^57, 58^. While these data imply that the Adriatic drainage deserves higher conservation attention than Danube drainage basin, our results suggest that the opposite may be true for subterranean and cryptic biodiversity. In South-East Slovenia and North-West Croatia (Danube basin), high cryptic diversity was found in several other subterranean species^59, 60^ as well as in epigean crayfish^61^ and fish^62^. Hence, we conclude that cryptic species may mask species richness patterns, that diversity of epigean fauna only poorly predicts the diversity of groundwater fauna, and that conservation of the two needs to proceed equally carefully.

Finally, we wish to discuss the potential problems that arise when discoveries of cryptic species by molecular taxonomic methods do not culminate in naming those species under the provisions of the Code. Naming new species is a major goal of traditional, morphology-based taxonomy, but obviously less so in modern taxonomy based on molecular data^9^. However, the lack of clear morphological diagnostic characters in no way implies the absence of biological or genetic barriers between populations, or that these populations do not differ in ecology, behaviour or evolutionary history. Our inability to diagnose species by traditional descriptions cannot be an excuse to ignore the part of evolutionary history that gave rise to forms too similar to be distinguished, or that had caused lineages to differ in several other characteristics except form. Molecular methods in most cases delimit species with higher accuracy than traditional, morphology-based methods and even provide statistical support for species hypotheses^2, 3^. Without the formal nomenclatural act, some of the most robust species hypotheses remain invisible to the broader biological community. Molecular taxonomic methods are about to become a standard in biomonitoring schemes (COST action CA15219 http://www.cost.eu/COST_Actions/ca/CA15219), and detecting species through their DNA trace in the environment (e-DNA) is becoming an increasingly important tool in ecology and conservation biology^63^. All the more, the need for molecular diagnoses and for the delivery of the Linnaean names to newly discovered cryptic species will increase in the future. Species defined and described by means of DNA sequences can be monitored and studied individually with help of DNA barcodes^63^, and easily included in conservation planning based on various metrics including phylogenetic diversity or endemism-corrected phylogenetic diversity^64, 65^.

Hence, assigning names in compliance with the Code based on molecular diagnoses alone is in our view a practice to be encouraged. The taxonomic infrastructure and rules have already been adopted^66^. However, we remain conservative regarding the species concept and amount of evidence required. More rigorous, rather than looser standards should be applied when relying on DNA data alone.

## Materials and Methods

### Sampling and DNA isolation

Based on information obtained from the database SubBioDB (http://subbio.net/db/) we selected 64 caves and springs in Slovenia and North-West Croatia, covering the entire range of *N. stygius sensu lato*. From these sites, 104 individuals were collected (Fig. 1), preserved in 96% ethanol and deposited in the Zoological Collection of the Department of Biology, Biotechnical faculty, University of Ljubljana, Slovenia. Information on collected specimens, including geographic coordinates, specimen vouchers, accession numbers and morphological species designation are available in Supplementary Information 1 (Table S1).

Genomic DNA was extracted from one of the pereopods using the GeneElute Mammalian Genomic DNA Miniprep Kit (Sigma-Aldrich). The remaining animal was deposited in the collection. We amplified nuclear DNA (nDNA) – two parts of the 28S rRNA gene (28S rRNA), internal transcribed spacer I and II (ITS I, II), histone 3 subunit A (H3) – and the mitochondrial (mtDNA) cytochrome oxidase I (COI) gene. A list of primers and PCR amplification programs used is available in Supplementary Information 2 (Table S2). Exonuclease I and Fast AP Thermosensitive Alkaline Phosphatase (Thermo Fisher Scientific Inc., US) were used to purify PCR products. These were sequenced using amplification primers by Macrogen Europe (Amsterdam, Netherlands). Sequences were assembled and chromatograms were visually inspected in Geneious 8.0.4. (Biomatters Ltd, New Zealand). Possible base polymorphisms and intragenomic variants were treated as ambiguous nucleotides and coded by wobble symbols. They were aligned in MAFFT v7 (Katoh and Standley 2013) under the E-INS-i algorithm.

### Phylogenetic analyses

We compiled a sequence dataset of 162 *Niphargus* individuals that, in addition to specimens of *N. stygius sensu lato* contained representatives of 37 other species from all main *Niphargus* lineages^22, 23^. The analysis was based on concatenated alignments of the 28 S, ITS, H3 and COI genes. Phylogenetic relationships were reconstructed using Bayesian inference (BA) in MrBayes v3.2^67^ and maximum likelihood in RAxML^68^.

The best-fitted evolutionary model for each gene partition as well as the optimal partitioning scheme were chosen via PartitionFinder^69^. As COI and H3 are protein coding genes, separate evolutionary models were selected for each codon position. The selected models are presented in Supplementary Information 2 (Table S2). For Bayesian inference, two parallel Markov chain Monte Carlo (MCMC) searches with four cold chains each were run for 5 million generations in MrBayes v3.2. Every 200^th^ generation was sampled, and the first 25% of the sampled trees were discarded as a burn-in, while the remaining trees were assembled into a majority-rule consensus tree with confidence assessed by posterior probabilities (Fig. 2).

A maximum likelihood multilocus phylogenetic analysis with a 100-replicate thorough bootstrap analysis was run in RAxML 7.8.3. Gene partitions were taken over from the BA analysis and evolutionary models were set to GTR + G + I for all partitions. All analyses were run on the CIPRES Web portal (www.phylo.org)^70–72^.

### Species delimitation procedures

Species hypotheses can be inferred using tree-based, distance based and multilocus coalescence allele sharing methods^3^. In this study, species were delineated using two tree-based and one distance-based species delimitation approaches. In the first step we performed unilocus delimitation using a Poisson Tree Processes model (PTP)^24^. PTP is a phylogeny-based species delimitation method, based on the assumption that intra and inter-specific nucleotide substitution levels differ notably and can be modelled as two independent Poisson processes. The analyses were performed on the COI alignment counting 117 niphargid mitochondrial COI sequences comprising herein studied and 37 other nominal species (Supplementary Information 1, Table S1). Phylogenetic relationships among these taxa were estimated in a separate MrBayes analysis; the settings used are the same as described above. The resulting consensus tree was then used to run the Bayesian implementation of Poisson tree processes (bPTP)^24^ analysis on the species delimitation server http://species.h-its.org/. Bayesian posterior probabilities for tentative species were acquired after running 500,000 generations, sampling every 100 generation, and discarding the first 20% of the samples as a burn-in.

In the multilocus approach, we used the multilocus coalescence delimitation method implemented in Bayesian Phylogenetics & Phylogeography 3.1 (BPP)^25^. As the *N. stygius* *s. lat*. species complex turned out to be non-monophyletic, we ran this analysis separately for each of the four clades containing species from this complex. The recent version of BPP does not require a user specified guiding tree^25^. Bayesian posterior probabilities for alternative species hypotheses were estimated via two alternative reversible-jump Markov chain Monte Carlo (rjMCMC, algorithms 0 and 1) searches on the multilocus molecular dataset with nearest neighbour interchange (NNI). Populations assigned to distinct evolutionary units were tested within a multilocus species delimitation framework, returning posterior probabilities for different number of species.

Ambiguities and missing data were excluded from the multilocus dataset. The Reverse jump MCMC was run for 30,000 generations. Every fifth generation was sampled, and the first 5,000 generations were omitted from subsequent analyses. Fine tuning parameters, heredity scalar and locus rate were estimated during the run. Different settings for ancestral population size (θ) and root age (τ) were set according to Leache and Fujita^73^: (i) θ = 2, 2000 and τ = 2, 2000, matching small ancestral population sizes and shallow divergences, (ii) θ = 1, 10 and τ = 1, 10, matching large ancestral population sizes and deep divergences, and (iii) θ = 1, 10 and τ = 1, 2000, matching large ancestral population sizes and shallow divergences. Each run was repeated twice to confirm the consistency of the resulting output.

In addition, we employed a distance-based delimitation approach using two, empirically determined thresholds that conservatively identify species boundaries^26, 27^. We first checked whether hypothetical species as identified using PTP and BPP diverged more than 16% in their patristic COI distances. The same alignment as for the PTP analysis was used to obtain a haplotype based phylogeny in PhyML^74^. The phylogeny was calculated under the GTR + G + I model of evolution with 4 substitution rate categories and a gamma shape parameter (α) of 0.397 as well as a proportion of invariant sites (0.399) estimated via maximum likelihood in PhyML. Patristic distances were extracted from the resulting phylogeny using the R package ape (version 3.4)^75^. Cryptic species were delimited using the R package cluster (version 2.0.4)^76^. Second, we checked whether hypothetical species diverged more than 4% in their pairwise Kimura-two-Parameter (K2P) distances^27^. K2P distances were calculated from the same dataset using the R package *adhoc* ^77^.

### Species richness, range size and evolutionary distinctness

Species richness is often used in nature conservation to assess the relative importance of sites or areas. We explored changes in species richness on a level of a single cave, on a level of drainage system and at national level.

In addition, we used two elementary indices, often combined in conservation biology to more complex metrics^32, 33^: range size as a measure of endemism, and evolutionary distinctness as a measure of phylogenetic uniqueness^32^. All collecting sites were spatially geocoded and mapped. Species range sizes were estimated in ArcGis 10.1 (ESRI) as minimum convex polygons.

In order to assess the phylogenetic uniqueness of the cryptic species, we calculated the evolutionary distinctness index (ED^32^) using the Tuatara 1.01 module^78^ in Mesquite 3.04^79^. The well-known ED is a measure of a species terminal branch-length, corrected for the species richness of the clade the species belongs to, i.e., species on longer branches with fewer congeners receives higher ED value than species within species rich radiations^32^. The virtue of this index is that it applies to individual species and warrants between-species comparisons.

### Species diagnoses

According to the Code, every species description needs to be supplemented with a diagnosis. In order to diagnose our cryptic species, we employed a two-step procedure. In the first step we identified morphological traits used in the diagnosis of *N. stygius* *s. lat*. as proposed by S. Karaman (1952), and provisionally identified specimens (not always possible).

In the second step we applied molecular diagnoses to individual species. Several authors discussed and described species using only molecular means^17–19^. We followed recent recommendations^17, 18^ and recently described protocols^18^. We used the Character Attribute Organization System (CAOS) software to determine diagnostic nucleotides^36^. For diagnoses we considered single nucleotides (character attributes, CA) present in all members of the monophyletic clades but absent from other clades (so called pure CA). CAOS identifies diagnostic combination of CA from alignments taking into account the hierarchic relationships of the species. For diagnostic purposes, we were interested only in species that cannot be identified on the basis of morphology alone. For those species, the morphological diagnosis of the complex as whole applies. From the molecular diagnosis we omitted all species that are not part of the focal species complex. Alignments of the three genes (28 S, COI and ITS) used in the analyses CAOS were constructed as described above using the subset of species we were interested in. The tree topology was obtained by pruning the phylogenetic tree presented in Fig. S1. In order to assure repeatability of results, the alignments are available in Dryad repository (doi:10.5061/dryad.6rs6q).

## Electronic supplementary material

Supplementary Information
