# Supplementary material for: The importance of naming cryptic species and the conservation of endemic subterranean amphipods
Source: Sci Rep. 2017 Jun 13;7:3391. doi: 10.1038/s41598-017-02938-z (PMC5469755; doi:10.1038/s41598-017-02938-z)
Supplement: Supplementary file 1 — Supplementary Information [file 41598_2017_2938_MOESM1_ESM.pdf]

The importance of naming cryptic species and the conservation of endemic subterranean amphipods

Supplementary Information

Teo Delić, Peter Trontelj, Michal Rendoš, Cene Fišer

Contents

*Supplementary Information 1***.** List of samples

*Supplementary Information 2.*Details on molecular analyses [supplementary information to Materials and Methods and Results, including Tables S2-S4, and Figure S1]

Supplementary Information 3. Morphological diagnosis of the complex, cryptic nature of the complex, etymology and holotype information. [including Table S5]

References

Supplementary Material 1. List of samples

Table S1. List of samples, localities and GenBank accession numbers

			Coordinates (wgs84)		Markers used			
**Species name**	**Voucher**	**Locality**	**longitude**	**latitude**	**28S**	**H3**	**COI**	**ITS**
N. aberrans	NA025	Ljubljanica freatik, Sneberje, Ljubljana	14.57311	46.08042	EF617260	NA	NA	NA
N. aitolosi	NA081	Lisimachia, Klisorevmata, Agrinio	21.36557	38.55653	EU693310	KP133142	NA	NA
N. arbiter	NA050	Izvir v luki, Vrbnik, Krk	14.67818	45.07839	EF617286	KR905885	NA	NA
N. arbiter	NA052	Tounjčica špilja, Tounj, Ogulin	15.32240	45.24871	EF617287	JQ815482	KR905787	KR905834
N. brachytelson	NA071	Lukova jama pri Zdihovem, Suhor, Kočevje	14.89410	45.52580	EU693293	JQ815489	KR905797	NA
N. brixianus	NC022	Grotta sotto la Cornabusa, Valpiana, Valle Imagna	9.53024	45.79816	KX379005	KX379050	KX379113	NA
N. carniolicus	NA017	Jama pod gradom Luknja, Prečna, Novo mesto	15.09978	45.81841	EF617252	JQ815525	KR905776	NA
N. caspary	NA073	Tuebingen, Tuebingen	9.05910	48.52136	EU693291	KJ566712	NA	NA
N. chagankae sp. n.	NA523	Jama v Kamnolomu, Vinica, Črnomelj	15.24431	45.45443	JQ815441	JQ815498	KR905828	KY617742
N. chagankae sp. n.	NB626	Dakina jama, Sekulići, Ozalj	15.30719	45.73133	NA	KY617577	NA	NA
N. chagankae sp. n.	NB792	Čaganka, Miklarji, Črnomelj	15.08215	45.54989	KY617397	KY617585	KY617497	KY617687
N. chagankae sp. n.	NB793	Čaganka, Miklarji, Črnomelj	15.08215	45.54989	KY617398	KY617586	KY617498	KY617688
N. chagankae sp. n.	NB794	Čaganka, Miklarji, Črnomelj	15.08215	45.54989	KY617399	KY617587	KY617499	KY617689
N. chagankae sp. n.	NB801	Zagorska peć, Zagorje, Ogulin	15.21990	45.19685	KY617406	KY617594	KY617506	KY617696
N. chagankae sp. n.	NB802	Zagorska peć, Zagorje, Ogulin	15.21990	45.19685	KY617407	KY617595	KY617507	NA
N. chagankae sp. n.	NB803	Zagorska peć, Zagorje, Ogulin	15.21990	45.19685	KY617408	KY617596	KY617508	KY617697
N. chagankae sp. n.	NB804	Špilja pod Kovačevcem, Bandino selo, Blagaj	15.57886	45.21826	KY617409	KY617597	KY617509	NA
N. chagankae sp. n.	NB805	Špilja pod Kovačevcem, Bandino selo, Blagaj	15.57886	45.21826	KY617410	KY617598	KY617510	NA
N. chagankae sp. n.	NB855	Kuštrovka, Ponikve, Ogulin	15.21992	45.34588	KY617419	KY617603	KY617519	KY617704
N. chagankae sp. n.	NB856	Kuštrovka, Ponikve, Ogulin	15.21992	45.34588	KY617420	KY617604	KY617520	KY617705

N. chagankae sp. n.	NB860	Dolača, Drašći vrh, Žumberak	15.47821	45.74012	KY617424	KY617608	NA	NA
N. chagankae sp. n.	NB861	Dolača, Drašći vrh, Žumberak	15.47821	45.74012	KY617425	KY617609	NA	NA
N. chagankae sp. n.	NB862	Dolača, Drašći vrh, Žumberak	15.47821	45.74012	KY617426	KY617610	NA	NA
N. chagankae sp. n.	NB931	Vodena jama, Kolići, Generalski stol	15.32199	45.34069	KY617446	KY617630	NA	KY617723
N. chagankae sp. n.	NC086	Dakina jama, Sekulići, Ozalj	15.30719	45.73133	KY617454	KY617639	NA	NA
N. costozzae	NA074	Covolo della Guerra, Longare, Vicenza	11.60000	45.48333	EU693294	KJ566713	KX379128	NA
N. cvajcki sp. n.	NA674	Velika jama nad Trebnjem, Trebnje	15.00723	45.89989	KY617371	KY617562	NA	KY617671
N. cvajcki sp. n.	NA675	Velika jama nad Trebnjem, Trebnje	15.00723	45.89989	KY617372	KY617563	NA	KY617672
N. cvajcki sp. n.	NA676	Velika jama nad Trebnjem, Trebnje	15.00723	45.89989	KY617373	KY617564	NA	KY617673
N. cvajcki sp. n.	NB795	Šimenkova jama, Gradišče, Stična	14.81691	45.96071	KY617400	KY617588	KY617500	KY617690
N. cvajcki sp. n.	NB796	Šimenkova jama, Gradišče, Stična	14.81691	45.96071	KY617401	KY617589	KY617501	KY617691
N. cvajcki sp. n.	NB797	Šimenkova jama, Gradišče, Stična	14.81691	45.96071	KY617402	KY617590	KY617502	KY617692
N. cvajcki sp. n.	NB798	Rivčja jama, Reberce, Žužemberk	14.87005	45.85604	KY617403	KY617591	KY617503	KY617693
N. cvajcki sp. n.	NB799	Rivčja jama, Reberce, Žužemberk	14.87005	45.85604	KY617404	KY617592	KY617504	KY617694
N. cvajcki sp. n.	NB800	Rivčja jama, Reberce, Žužemberk	14.87005	45.85604	KY617405	KY617593	KY617505	KY617695
N. cvajcki sp. n.	NB914	Šolnovo brezno, Prevole, Žužemberk	14.85012	45.77781	KY617433	KY617617	KY617529	KY617711
N. cvajcki sp. n.	NB915	Šolnovo brezno, Prevole, Žužemberk	14.85012	45.77781	KY617434	KY617618	KY617530	KY617712
N. cvijici	NA147	Ravno, Popovo polje	17.98643	42.89404	JQ815554	JQ815516	KR905819	NA
N. dalmatinus	NA060	Biba, Vrana, Pakoštane	15.57059	43.94897	EF617296	JQ815484	KR905790	KY617657
N. dancaui	NA152	Valeni, Ploiesti, Prahova	26.03001	44.94002	KJ566693	KJ566720	KR905821	NA
N. dobati	NA013	Mali naravni most, Rakov škocjan, Rakek	14.30554	45.79097	EF617247	JQ815499	KR905774	NA
N. elegans	NA061	San Pancrazio, Verona	11.02106	45.42383	EF617297	JQ815485	KR905791	KY617658
N. elegans	NA518	Reka Arno, Firenze	11.25522	43.76780	NA	KY617561	KY617482	NA
N. fongi	NA018	Dolga jama pri Koblarjih, Koblarji, Kočevje	14.83005	45.71220	EF617253	JQ815472	NA	NA
N. frasassianus	NA	Frassasi, Genga, Ancona	12.96194	43.40083	GU973411	NA	GU973135	NA

N. goricae sp. n.	NA085	Vodnjak v Framu, Fram, Maribor	15.63948	46.45543	EU693305	KR905888	KR905803	KY617654
N. goricae sp. n.	NB932	Vodnjak v Ješenci, Ješenca, Rače	15.65074	46.44123	KY617447	KY617631	KY617534	KY617724
N. goricae sp. n.	NC091	Vodnjak v Senarci, Senarca, Lenart	15.88860	46.55451	KY617459	KY617643	KY617544	KY617732
N. goricae sp. n.	NC092	Vodnjak v Čakovi, Čakova, Cerkvenjak	16.01478	46.58349	NA	NA	KY617545	NA
N. goricae sp. n.	NC093	Vodnjak v Čakovi, Čakova, Cerkvenjak	16.01478	46.58349	KY617460	NA	KY617546	NA
N. goricae sp. n.	NC094	Vodnjak v Čakovi, Čakova, Cerkvenjak	16.01478	46.58349	KY617461	KY617644	KY617547	KY617733
N. goricae sp. n.	NC095	Luža v Lastomercih, Lastomerci, Gornja Radgona	15.96048	46.63430	KY617462	KY617645	KY617548	KY617734
N. goricae sp. n.	NC096	Luža v Lastomercih, Lastomerci, Gornja Radgona	15.96048	46.63430	KY617463	KY617646	KY617549	KY617735
N. goricae sp. n.	NC097	Luža v Lastomercih, Lastomerci, Gornja Radgona	15.96048	46.63430	KY617464	KY617647	KY617550	KY617736
N. gottscheeanensis sp. n.	NA516	Izvir SW od Sušja, Sušje, Ribnica	14.68105	45.75300	KY617466	KY617648	KY617551	KY617738
N. gottscheeanensis sp. n.	NA706	Podpeška jama, Podpeč, Dobrepolje	14.68632	45.83926	KY617377	KY617567	KY617485	KY617677
N. gottscheeanensis sp. n.	NA707	Podpeška jama, Podpeč, Dobrepolje	14.68632	45.83926	KY617378	KY617568	KY617486	KY617678
N. gottscheeanensis sp. n.	NB789	Jama v Kamnolomu, Vinica, Črnomelj	15.24431	45.45443	KY617394	NA	KY617496	KY617686
N. gottscheeanensis sp. n.	NB790	Židovske kuće, Budinjak, Žumberak	15.50069	45.78829	KY617395	KY617583	NA	NA
N. gottscheeanensis sp. n.	NB844	Željnske jame, Željne, Kočevje	14.88488	45.65427	KY617412	KY617600	KY617512	KY617699
N. gottscheeanensis sp. n.	NB845	Željnske jame, Željne, Kočevje	14.88488	45.65427	KY617413	KY617601	KY617513	KY617700
N. gottscheeanensis sp. n.	NB925	Izviri NW od Kočevske reke, Kočevska reka, Kočevje	14.78968	45.58124	KY617440	KY617624	KY617533	KY617717
N. gottscheeanensis sp. n.	NB926	Izviri NW od Kočevske reke, Kočevska reka, Kočevje	14.78968	45.58124	KY617441	KY617625	NA	KY617718
N. gottscheeanensis sp. n.	NB927	Izviri NW od Kočevske reke, Kočevska reka, Kočevje	14.78968	45.58124	KY617442	KY617626	NA	KY617719
N. gottscheeanensis sp.	NC076	Izvir ob Obrhu, Mirtoviči, Osilnica	14.77907	45.51913	KY617449	KY617634	KY617537	KY617726

n.								
N. hadzii	NA082	Izvir pod orehom, Verd, Vrhnika	14.29966	45.95250	EU693301	KR905887	KR905800	KY617660
N. illidzensis	NA084	Vrelo Bosne, Ilidža, Sarajevo	18.26724	43.81927	EU693304	JQ815491	KR905802	KY617661
N. iskae sp. n.	NB619	Mačji rep, Škrabče, Nova vas	14.53212	45.81361	KY617382	KY617571	KY617490	NA
N. iskae sp. n.	NC087	Mačji rep, Škrabče, Nova vas	14.53212	45.81361	KY617455	KY617640	KY617542	KY617730
N. iskae sp. n.	NC088	Mačji rep, Škrabče, Nova vas	14.53212	45.81361	KY617456	KY617641	KY617543	KY617731
N. kapelanus sp. n.	NB625	Špilja pod Mačkovom dragom, Vrelo, Jasenak	15.02382	45.26099	KY617387	KY617576	KY617494	NA
N. karamani	NB933	Vodnjak v Pečkah, pečke, Majšperk	15.67440	46.33390	NA	KY617632	KY617535	NA
N. kenki	NA086	Izvir v Sodni vasi, Sodna vas, Podčetrtek	15.59486	46.17618	KR905869	KY617556	KR905804	KY617662
N. kenki	NA087	Polje of Sotli, Bistrica ob Sotli, Brežice	15.66410	46.06790	EU693306	NA	KY617476	KY617663
N. kenki	NA201	Marijino brezno, Lubnik, Škofja loka	14.29633	46.16465	KY617368	KY617559	KY617479	KY617669
N. kenki	NA236	Ljubljanica freatik, Tomačevo, Ljubljana	14.54057	46.08281	KY617369	KY617560	KY617480	KY617670
N. kenki	NA239	Ljubljanica freatik, Sneberje, Ljubljana	14.57312	46.08043	KY617370	NA	KY617481	NA
N. kolombatovici	NA964/NA963	Žira, Dobri do, Popovo polje	17.88478	42.91670	JQ815553	JQ815522	KT007386	NA
N. kordunensis sp. n.	NB623	Matešička špilja, Matešići, Slunj	15.61389	45.10808	KY617386	KY617575	NA	NA
N. kordunensis sp. n.	NC089	Matešička špilja, Matešići, Slunj	15.61389	45.10808	KY617457	NA	NA	NA
N. kordunensis sp. n.	NC090	Matešička špilja, Matešići, Slunj	15.61389	45.10808	KY617458	KY617642	NA	NA
N. krameri	NA040	Stranski pritok Fojbe, Šestani, Pazin	13.98650	45.24621	EF617275	JQ815503	NA	NA
N. labacensis	NA022	Ljubljanica freatik, Tomačevo, Ljubljana	14.54057	46.08280	EF617257	JQ815474	KR905777	NA
N. lessiniensis	NA064	Grotta dell Aqua, Ponte di Veja, Verona,ITA	10.97021	45.60815	EF617300	JQ815488	KR905794	NA
N. likanus	NB917	Đula-Medvednica, Ogulin	15.22399	45.26704	KY617435	KY617619	NA	KY617713
N. likanus	NB918	Đula-Medvednica, Ogulin	15.22399	45.26704	KY617436	KY617620	NA	KY617714
N. likanus	NB922	Izvir v Juzbašićih, Juzbašići, Tounj	15.42788	45.19763	KY617437	KY617621	KY617531	KY617715
N. likanus	NB923	Izvir v Juzbašićih, Juzbašići, Tounj	15.42788	45.19763	KY617438	KY617622	KY617532	KY617716

N. likanus	NB924	Izvir v Juzbašićih, Juzbašići, Tounj	15.42788	45.19763	KY617439	KY617623	NA	NA
N. longicaudatus [Cres]	NA006	Retec, Lubenice, Cres	14.33641	44.88768	EF617240	KJ566705	KR905772	NA
N. longicaudatus [N]	NA007	Monte Faito, Vasola, Napoli	14.46006	40.66946	EF617241	JQ815469	NA	NA
N. longidactylus	NA021	Ljubljanica freatik, Sneberje, Ljubljana	14.57311	46.08042	EF617256	JQ815473	NA	NA
N. longiflagellum	NA093	Podpeška jama, Podpeč, Dobrepolje	14.68632	45.83926	EU693311	JQ815520	KR905805	KR905840
N. malagorae sp. n.	NB621	Vančeva jama, Koblarji, Kočevje	14.82263	45.70310	KY617384	KY617573	KY617492	KY617682
N. malagorae sp. n.	NB857	Mivčje jama, Gornje Lepovčje, Ribnica	14.73370	45.75350	KY617421	KY617605	NA	NA
N. malagorae sp. n.	NB858	Mivčje jama, Gornje Lepovčje, Ribnica	14.73370	45.75350	KY617422	KY617606	KY617521	NA
N. malagorae sp. n.	NB859	Mivčje jama, Gornje Lepovčje, Ribnica	14.73370	45.75350	KY617423	KY617607	KY617522	NA
N. malagorae sp. n.	NC083	Vančeva jama, Koblarji, Kočevje	14.82263	45.70310	KY617452	KY617637	KY617540	NA
N. malagorae sp. n.	NC084	Vančeva jama, Koblarji, Kočevje	14.82263	45.70310	KY617453	KY617638	KY617541	KY617729
N. novomestanus	NA096	Tominčev studenc, Žužemberk	14.96855	45.79628	EU693314	JQ815509	KR858496	KY617740
N. novomestanus	NA097	Tominčev studenc, Žužemberk	14.96855	45.79628	KY617469	KY617650	KY617552	KY617741
N. novomestanus	NA098	izvir Gabrovčec, Gabrovčec, Krška vas	14.80170	45.88089	KY617364	KY617554	KY617473	KY617653
N. novomestanus	NA131	Jama pod gradom Luknja, Prečna, Novo mesto	15.09978	45.81841	KY617365	KY617555	KY617474	KY617655
N. novomestanus	NA178	Sela pri Straži, Straža, Novo mesto	15.09644	45.78730	KY617467	KY617649	NA	NA
N. novomestanus	NA708	Vodanjak v Podgori, Podgora, Prečna	15.10222	45.80784	KY617379	KY617569	KY617487	NA
N. novomestanus	NB617	Rupa na Brodu, Šmihel, Novo mesto	15.14429	45.78885	KY617381	KY617570	KY617489	KY617680
N. novomestanus	NB786	Vodanjak v Podgori, Podgora, Prečna	15.10222	45.80784	KY617391	NA	NA	KY617684
N. novomestanus	NB787	Vodanjak v Podgori, Podgora, Prečna	15.10222	45.80784	KY617392	KY617581	NA	KY617685
N. novomestanus	NB788	Vodanjak v Podgori, Podgora, Prečna	15.10222	45.80784	KY617393	KY617582	KY617495	NA
N. novomestanus	NB791	Židovske kuće, Budinjak, Žumberak	15.50069	45.78829	KY617396	KY617584	NA	NA

N. pachytelson	NA100	Podpeška jama, Podpeč, Dobrepolje	14.68632	45.83926	EU693316	JQ815511	KR905809	KR905841
N. pasquinii	NA010	Sorgenti san Vittorino, San Vittorino, Rieti	12.99077	42.37629	EF617244	JQ815471	KR905773	NA
N. pectinicauda	NA023	Ljubljanica freatik, Tomačevo, Ljubljana	14.54057	46.08280	EF617258	JQ815475	KR905778	NA
N. podpecanus	NA101	Podpeška jama, Podpeč, Dobrepolje	14.68632	45.83926	EU693317	JQ815512	KR905810	NA
N. podpecanus	NA693	Slugova jama, Golobinjek, Dolenjske toplice	15.05148	45.84125	KY617374	NA	KY617483	KY617674
N. podpecanus	NA704	Izvir pri črpališčuvenska vas, Kočevje	14.83022	45.66765	KY617375	KY617565	KY617484	KY617675
N. podpecanus	NA705	Izvir pri črpališčuvenska vas, Kočevje	14.83022	45.66765	KY617376	KY617566	NA	KY617676
N. podpecanus	NB620	Izvir ob Obrhu, Mirtoviči, Osilnica	14.77907	45.51913	KY617383	KY617572	KY617491	KY617681
N. podpecanus	NB622	Krška jama, Gradiček, Krška vas	14.77126	45.88999	KY617385	KY617574	KY617493	KY617683
N. podpecanus	NB843	Željnske jame, Željne, Kočevje	14.88488	45.65427	KY617411	KY617599	KY617511	KY617698
N. podpecanus	NB863	Črničkova jama, Stavča vas, Žužemberk	14.96267	45.80824	KY617427	KY617611	KY617523	KY617706
N. podpecanus	NB864	Črničkova jama, Stavča vas, Žužemberk	14.96267	45.80824	KY617428	KY617612	KY617524	KY617707
N. podpecanus	NB865	Črničkova jama, Stavča vas, Žužemberk	14.96267	45.80824	KY617429	KY617613	KY617525	KY617708
N. podpecanus	NB910	Lučka jama, Luče, Grosuplje	14.72514	45.91778	KY617430	KY617614	KY617526	NA
N. podpecanus	NB911	Lučka jama, Luče, Grosuplje	14.72514	45.91778	KY617431	KY617615	KY617527	KY617709
N. podpecanus	NB912	Lučka jama, Luče, Grosuplje	14.72514	45.91778	KY617432	KY617616	KY617528	KY617710
N. podpecanus	NB928	Jama v Peklu, Rajndol, Kočevje	14.97470	45.56470	KY617443	KY617627	NA	KY617720
N. podpecanus	NB929	Jama v Peklu, Rajndol, Kočevje	14.97470	45.56470	KY617444	KY617628	NA	KY617721
N. podpecanus	NB930	Jama v Peklu, Rajndol, Kočevje	14.97470	45.56470	KY617445	KY617629	NA	KY617722
N. podpecanus	NC075	Izvir ob Obrhu, Mirtoviči, Osilnica	14.77907	45.51913	KY617448	KY617633	KY617536	KY617725
N. podpecanus	NC081	Krška jama, Gradiček, Krška vas	14.77126	45.88999	KY617450	KY617635	KY617538	KY617727
N. podpecanus	NC082	Krška jama, Gradiček, Krška vas	14.77126	45.88999	KY617451	KY617636	KY617539	KY617728
N. parapupetta	NA102	Ljubljanica freatik, Tomačevo, Ljubljana	14.54057	46.08280	EU693318	KJ566717	NA	NA

N. puteanus	NA066	Gasthof Zur Walba, Pentling, Regensburg	12.03579	48.97457	EF617302	KJ566709	KR905795	NA
N. rejici	NA048	Podpeško jezero, Podpeč, Ig	14.43211	45.96840	EF617283	JQ815481	KR905785	KR905833
N. slovenicus	NA106	Stražišče, Kranj	14.33766	46.22713	EU693322	JQ815493	KR905813	KY617666
N. sphagnicolus	NA035	Mostec, Rožnik, Ljubljana	14.47319	46.06115	EF617270	NA	KR858495	NA
N. spoeckeri	NA108	Pivka jama, Veliki otok, Postojna	14.20451	45.80459	EU693324	JQ815513	KR905814	KY617667
N. spoeckeri	NA179	Zelške jame, Zelše, Cerknica	14.30349	45.79065	KY617367	KY617558	KY617478	NA
N. stygius	NA110	Jelenska jama, Borovnica, Vrhnika	14.35549	45.91790	KR905870	KR905890	KR905815	NA
N. stygius	NA123	Predjamski sistem, Bukovje, Postojna	14.12660	45.81567	EU693325	JQ815515	KR858498	NA
N. stygius	NA678	Vogrščček, Avče, Nova Gorica	13.71233	46.12534	KY617472	NA	NA	KY617744
N. stygius	NA733	Šumnik, Lepena, Bovec	13.64978	46.31890	KY617468	NA	NA	KY617739
N. stygius	NA735	Marijino brezno, Lubnik, Škofja loka	14.29633	46.16465	KY617366	NA	NA	KY617656
N. stygius	NA758	Bunker in Visintini, Dobredob, Gorica	13.56694	45.86639	KY617471	NA	NA	KY617743
N. stygius	NA788	Studenec nad sv. Marjeto, Palčje, Pivka	14.26950	45.68906	KY617465	NA	NA	KY617737
N. stygius	NA873	Čendova jama, Klavže, Šentviška gora	13.82258	46.14960	KY617363	NA	NA	KY617652
N. timavi	NA114	Labodnica, Trebče, Trst	13.82859	45.68448	EU693327	JQ815495	KR858497	NA
N. tridentinus	NA063	Grotta Bus Pursi, Lumezzane, Brescia	10.27419	45.64601	EF617299	JQ815487	KR905793	NA
N. vinodolensis	NA062	Cerovići, Bačići, Novi Vinodolski	14.64687	45.23345	EF617298	JQ815486	KR905792	KY617659
N. wolfi	NA015	Križna jama, Bločice, Lož	14.46727	45.74519	EF617250	JQ815500	KR905775	NA
N. zagrebensis	NA059	Gadina, Loka, Črnomelj	15.18202	45.56461	EF617295	KR905886	KR905789	NA
N. zagrebensis	NA117	Čučerje, Dubrava, Zagreb	16.06069	45.89635	EU693330	KY617557	KY617477	KY617668
N. zagrebensis	NA709	Kočevske poljane, Dolenjske toplice	15.05483	45.72460	KY617380	NA	KY617488	KY617679
N. zagrebensis	NB783	Izvir v Jurišah, Juriše, Ozalj	15.47533	45.56973	KY617388	KY617578	NA	NA
N. zagrebensis	NB784	Izvir v Jurišah, Juriše, Ozalj	15.47533	45.56973	KY617389	KY617579	NA	NA
N. zagrebensis	NB785	Izvir v Jurišah, Juriše, Ozalj	15.47533	45.56973	KY617390	KY617580	NA	NA
N. zagrebensis	NB846	Božakovska jama, Božakovo, Metlika	15.37489	45.64777	KY617414	NA	KY617514	NA

N. zagrebensis	NB847	Božakovska jama, Božakovo, Metlika	15.37489	45.64777	KY617415	NA	KY617515	KY617701
N. zagrebensis	NB848	Božakovska jama, Božakovo, Metlika	15.37489	45.64777	KY617416	NA	KY617516	KY617702
N. zagrebensis	NB850	Gadina, Loka, Črnomelj	15.18202	45.56461	KY617417	KY617602	KY617517	KY617703
N. zagrebensis	NB851	Gadina, Loka, Črnomelj	15.18202	45.56461	KY617418	NA	KY617518	NA

Supplementary Material 2. Details on molecular analyses

[Supplementary information to Materials and Methods and Results]

Materials and Methods

For PCR amplification of the studied DNA sequences, we used the primers listed in Table S2. For Bayesian phylogenetic analyses, we selected evolutionary models by gene partitions as proposed using PartitionFinder[1]. The selected models are presented Table S2. Multilocus coalescence analysis using BPP was run under a number of conditions. The conditions and detailed results are listed in Table S3. In Fig. S1 we show the full phylogenetic tree with all individuals used in the analysis.

Supplementary Table S2. List of primers and selected evolutionary models.

Gene	Primers	Partition in concatenated dataset	Best Substitution Model
28S rRNA[2]	28S lev2-28S des2	1-902	K80+G+I
Histone H3[3]	H3aF2-H3aR2	903-1232\3	JC
		904-1232\3	JC
		905-1232\3	GTR+G
Cytochrome Oxidase I*[4,5]	LCO- COIspr1	1233-1799\3	SYM+G
		1234-1799\3	F81+G+I
		1235-1799\3	GTR+G
Internal Transcribed Spacer[6]	ITSf1-ITSr1	1800 - 5425	K80+G+I

*The COI gener was used in uni- and multilocus analyses. The selected evolutionary models were identical for both analyses.

Supplementary Table S3. Results of multilocus coalescence species delimitation analysis using the program BPP, indicating the probability for maximum number of species estimated under various settings. Three separate analyses were run, for each clade separately.

prior θ	prior τ	algorithm 0	algorithm 1
Niphargus karamani			
large pop., deep div. G (1, 10)	G (1, 10)	**1 (2 spp.)**	**1 (2 spp.)**
		0.00 (1 spp.)	0.00 (1 spp.)
small pop., shallow div. G (2, 2000)	G (2, 2000)	**0.9998 (2 spp.)**	**0.9996 (2 spp.)**
		0.0002 (1 spp.)	0.0004 (1 spp.)
large pop., shallow div. G (1, 10)	G (2, 2000)	**1 (2 spp.)**	**1 (2 spp.)**
		0.00 (1 spp.)	0.00 (1 spp.)
Niphargus kenki			
large pop., deep div. G (1, 10)	G (1, 10)	**0.84673 (2 spp.)**	**0.84983 (2 spp.)**
		0.15327 (1 spp.)	0.15017 (1 spp.)
small pop., shallow div. G (2, 2000)	G (2, 2000)	**0.99893 (2 spp.)**	**0.99433 (2 spp.)**
		0.00107 (1 spp.)	0.00567 (1 spp.)
large pop., shallow div. G (1, 10)	G (2, 2000)	**0.99527 (2 spp.)**	**0.99683 (2 spp.)**
		0.00473 (1 spp.)	0.00317 (1 spp.)
Niphargus novomestanus complex			
large pop., deep div. G (1, 10)	G (1, 10)	**0.42513 (19 spp.)**	**0.42527 (19 spp.)**
		0.36247 (18 spp.)	0.34827 (18 spp.)
		0.1637 (17 spp.)	0.16907 (17 spp.)
		0.04307 (16 spp.)	0.0503 (16 spp.)
		0.00563 (15 spp.)	0.00677 (15 spp.)
		/	0.00033 (14 spp.)
small pop., shallow div. G (2, 2000)	G (2, 2000)	**0.90763 (19 spp.)**	**0.84773 (19 spp.)**
		0.07187 (18 spp.)	0.14303 (18 spp.)
		0.0202 (17 spp.)	0.009 (17 spp.)
		0.0003 (16 spp.)	0.00023 (16 spp.)
large pop., shallow div. G (1, 10)	G (2, 2000)	**0.54687 (19 spp.)**	**0.64333 (19 spp.)**
		0.32733 (18 spp.)	0.27437 (18 spp.)
		0.10317 (17 spp.)	0.0679 (17 spp.)
		0.01923 (16 spp.)	0.0138 (16 spp.)
		0.00323 (15 spp.)	0.0006 (15 spp.)
		0.00017 (14 spp.)	/

Θ – population size; τ – time of divergence; Algorithm 0 and 1 – two alternative reversible-jump proposals used in multilocus species delimitation, the two algorithms differ in how they calculate ancestral Θ values.

Table S4. Pairwise COI gene distances in between studied Niphargus species. Above the diagonal are K2P distances (threshold 0.04), below patristic distances (threshold 0.16). Presented are mean values, minimum and maximum values are in brackets. Pairs below the thresholds[1] are presented in boldface.

	N. goricae	***N. karamani***	***N. cvajcki***	***N. spoeckeri***	***N. iskae***	***N. kapelanus***	***N. zagrebensis***	***N. likanus***	***N. brachytelson***	***N.malagorae***	***N. gottsch.***	***N. novomest.***	***N. podpecanus***	***N. chagankae***	***N.kenki***	***N. stygius***
N. goricae		0.096 (0.092-0.099)	0.193 (0.187-0.200)	0.200 (0.190-0.209)	0.212 (0.207-0.217)	0.164 (0.161-0.166)	0.174 (0.166-0.179)	0.167 (0.165-0.167)	0.191 (0.190-0.192)	0.195 (0.188-0.201)	0.174 (0.164-0.179)	0.185 (0.178-0.203)	0.188 (0.183-0.193)	0.217 (0.204-0.234)	0.190 (0.181-0.020)	0.198 (0.192-0.202)
N. karamani	0.163 (0.155-0.167)		0.179 (0.175-0.187)	0.176 (0.169-0.183)	0.192 (0.191-0.193)	0.175 (0.175-0.175)	0.173 (0.168-0.183)	0.161 (0.161-0.161)	0.183 (0.183-0.183)	0.188 (0.185-0.19)	0.163 (0.159-0.166)	0.170 (0.163-0.188)	0.179 (0.168-0.183)	0.193 (0.188-0.205)	0.164 (0.161-0.166)	0.194 (0.194-0.194)
N. cvajcki	0.441 (0.424-0.459)	0.458 (0.448-0.471)		0.156 (0.143-0.171)	0.175 (0.168-0.185)	0.154 (0.147-0.168)	0.165 (0.156-0.175)	0.163 (0.159-0.168)	0.1925 (0.189-0.200)	0.177 (0.172-0.18)	0.153 (0.145-0.161)	0.154 (0.145-0.169)	0.170 (0.158-0.192)	0.169 (0.163-0.176)	0.167 (0.154-0.189)	0.173 (0.168-0.184)
N. spoeckeri	0.491 (0.471-0.507)	0.508 (0.496-0.520)	0.506 (0.485-0.533)		0.085 (0.078-0.092)	0.129 (0.120-0.138)	0.146 (0.136-0.158)	0.117 (0.109-0.124)	0.145 (0.138-0.152)	0.145 (0.136-0.154)	0.113 (0.103-0.125)	0.123 (0.107-0.136)	0.123 (0.109-0.138)	0.156 (0.143-0.168)	0.147 (0.136-0.156)	0.159 (0.149-0.168)
N. iskae	0.497 (0.487-0.501)	0.513 (0.511-0.514)	0.511 (0.501-0.526)	**0.136 (0.122-0.149)**		0.141 (0.140-0.142)	0.152 (0.147-0.161)	0.130 (0.129-0.131)	0.135 (0.133-0.136)	0.163 (0.161-0.164)	0.128 (0.124-0.131)	0.133 (0.127-0.136)	0.122 (0.115-0.129)	0.161 (0.154-0.166)	0.134 (0.127-0.143)	0.151 (0.150-0.152)
N. kapelanus	0.471 (0.462-0.474)	0.487 (0.487-0.487)	0.486 (0.477-0.500)	0.266 (0.254-0.278)	0.271 (0.270-0.272)		0.125 (0.118-0.129)	0.124 (0.124-0.124)	0.138 (0.138-0.138)	0.131 (0.129-0.133)	0.123 (0.122-0.127)	0.125 (0.118-0.133)	0.119 (0.113-0.124)	0.167 (0.159-0.175)	0.135 (0.129-0.140)	0.140 (0.140-0.140)
N. zagrebensis	0.520 (0.507-0.533)	0.536 (0.531-0.546)	0.534 (0.521-0.559)	0.315 (0.298-0.337)	0.320 (0.314-0.331)	0.272 (0.267-0.282)		0.120 (0.118-0.122)	0.124 (0.12-0.136)	0.101 (0.096-0.111)	0.111 (0.102-0.116)	0.110 (0.100-0.116)	0.093 (0.083-0.107)	0.132 (0.116-0.150)	0.134 (0.118-0.152)	0.142 (0.140-0.147)
N. likanus	0.519 (0.510-0.522)	0.534 (0.534-0.534)	0.533 (0.524-0.547)	0.314 (0.302-0.326)	0.318 (0.317-0.319)	0.270 (0.270-0.270)	0.246 (0.241-0.256)		0.116 (0.116-0.116)	0.116 (0.114-0.120)	0.076 (0.073-0.082)	0.090 (0.079-0.103)	0.112 (0.105-0.118)	0.124 (0.118-0.129)	0.135 (0.133-0.138)	0.142 (0.142-0.142)
N. brachytelson	0.580 (0.567-0.579)	0.591 (0.591-0.591)	0.590 (0.581-0.604)	0.371 (0.359-0.383)	0.375 (0.374-0.376)	0.327 (0.327-0.327)	0.303 (0.298-0.313)	0.266 (0.266-0.266)		0.084 (0.082-0.085)	0.114 (0.111-0.116)	0.116 (0.109-0.130)	0.116 (0.111-0.127)	0.144 (0.132-0.152)	0.142 (0.133-0.152)	0.166 (0.166-0.166)
N.malagorae	0.552 (0.540-0.560)	0.568 (0.564-0.572)	0.566 (0.554-0.585)	0.347 (0.331-0.364)	0.352 (0.347-0.357)	0.304 (0.300-0.308)	0.279 (0.271-0.294)	0.243 (0.239-0.247)	**0.139 (0.135-0.143)**		0.103 (0.098-0.109)	0.109 (0.101-0.121)	0.100 (0.094-0.109)	0.131 (0.120-0.143)	0.150 (0.145-0.154)	0.165 (0.163-0.168)
N. gottsch.	0.529 (0.517-0.537)	0.545 (0.542-0.549)	0.543 (0.532-0.562)	0.324 (0.309-0.341)	0.329 (0.325-0.334)	0.281 (0.278-0.285)	0.256 (0.248-0.271)	0.220 (0.216-0.224)	0.257 (0.254-0.261)	0.234 (0.227-0.242)		0.060 (0.055-0.065)	0.089 (0.081-0.098)	0.114 (0.103-0.127)	0.140 (0.133-0.147)	0.154 (0.147-0.159)
N. novomest.	0.524 (0.507-0.535)	0.541 (0.532-0.548)	0.539 (0.522-0.560)	0.320 (0.299-0.339)	0.325 (0.315-0.333)	0.276 (0.268-0.283)	0.252 (0.239-0.269)	0.215 (0.207-0.222)	0.253 (0.244-0.260)	0.229 (0.217-0.241)	**0.115 (0.103-0.126)**		0.094 (0.085-0.105)	0.098 (0.083-0.116)	0.138 (0.131-0.147)	0.149 (0.138-0.156)
N. podpec.	0.562 (0.545-0.571)	0.578 (0.570-0.584)	0.577 (0.560-0.597)	0.357 (0.337-0.375)	0.362 (0.353-0.369)	0.314 (0.306-0.320)	0.289 (0.277-0.305)	0.253 (0.245-0.259)	0.290 (0.282-0.296)	0.267 (0.255-0.277)	0.223 (0.212-0.233)	0.218 (0.202-0.231)		0.113 (0.105-0.120)	0.147 (0.135-0.154)	0.142 (0.135-0.144)
N. chagankae	0.646 (0.612-0.675)	0.662 (0.637-0.688)	0.660 (0.627-0.700)	0.441 (0.404-0.479)	0.446 (0.420-0.473)	0.398 (0.373-0.423)	0.373 (0.343-0.409)	0.337 (0.311-0.362)	0.374 (0.349-0.400)	0.350 (0.322-0.381)	0.307 (0.278-0.337)	0.302 (0.269-0.335)	0.298 (0.265-0.330)		0.155 (0.145-0.166)	0.160 (0.151-0.163)
N.kenki	0.519 (0.503-0.527)	0.535 (0.528-0.539)	0.533 (0.518-0.552)	0.365 (0.346-0.382)	0.370 (0.362-0.376)	0.344 (0.337-0.349)	0.393 (0.382-0.408)	0.392 (0.385-0.396)	0.449 (0.442-0.453)	0.425 (0.415-0.434)	0.402 (0.392-0.411)	0.398 (0.383-0.410)	0.435 (0.420-0.446)	0.519 (0.487-0.550)		0.131 (0.124-0.136)
N. stygius	0.498 (0.489-0.501)	0.514 (0.514-0.514)	0.512 (0.503-0.526)	0.344 (0.332-0.356)	0.349 (0.347-0.350)	0.323 (0.323-0.323)	0.372 (0.367-0.382)	0.370 (0.370-0.370)	0.427 (0.427-0.427)	0.403 (0.400-0.408)	0.381 (0.378-0.385)	0.377 (0.368-0.384)	0.414 (0.406-0.420)	0.498 (0.473-0.524)	0.280 (0.273-0.284)	

[1] Species pairs below threshold are presented also in the main text as Fig.3.

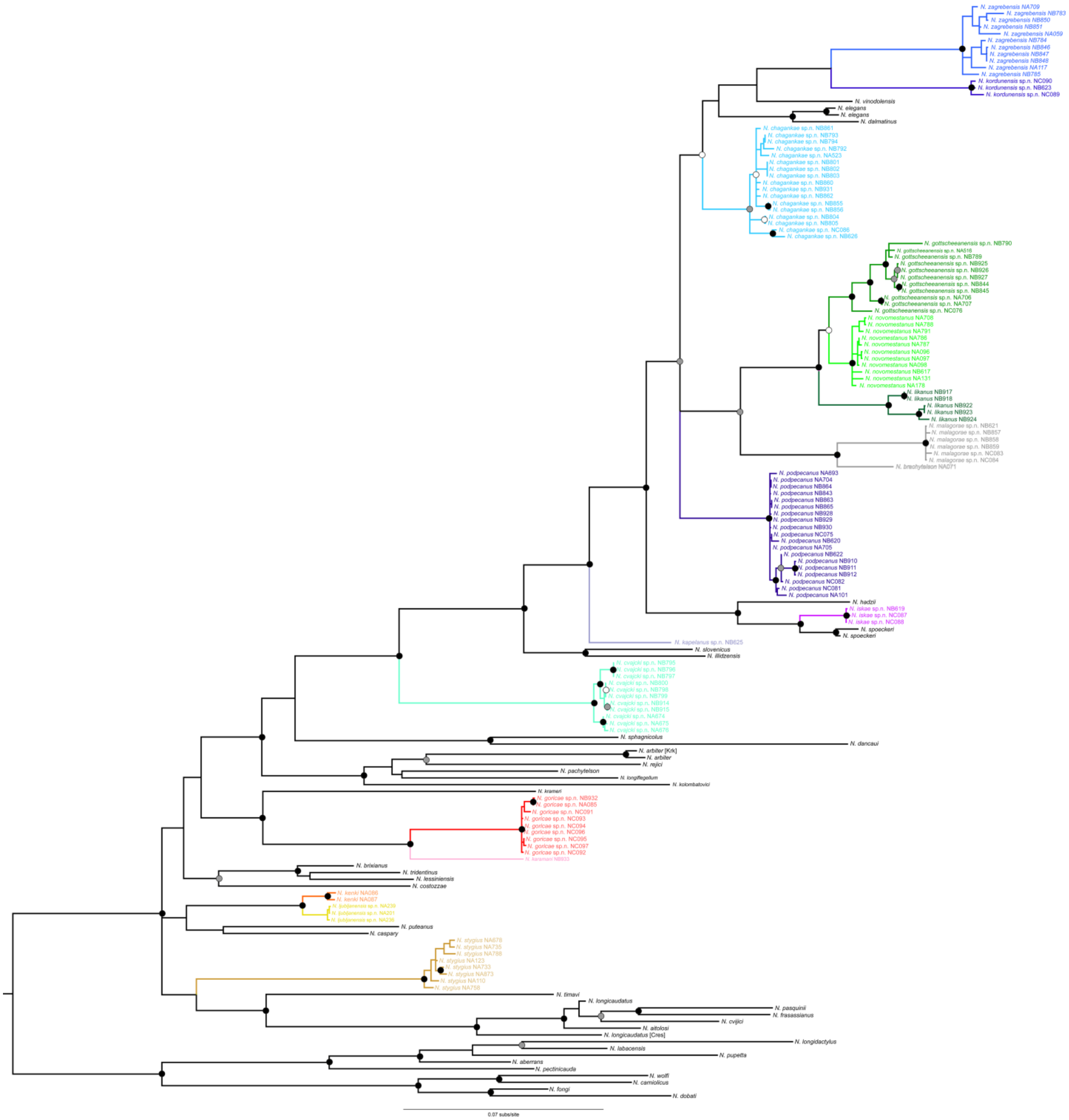

Supplementary Figure S1. Bayesian phylogenetic tree of all analyzed individuals; labels include specimen vouchers.

Supplementary Material 3. Morphological diagnosis of the complex, etymology and data about holotypes.

Morphological diagnosis of the *N. stygius* complex

Niphargus stygius is the type species of the genus *Niphargus*, described from the caves Postojnska jama and Predjama[7]. Being the first species described, it is one of the species that was repeatedly reported from all parts of the genus range, either as "*N. stygius*" or as some of its forms or subspecies. As the taxonomy of the genus developed, many of these forms or subspecies have been recognized as separate species.

Stanko Karaman was the first who realized that this species needs to be redefined, and in 1952 he published a revision of *N. stygius* from the area of Slovenia and Croatia. Based on his extensive analyses, he suggested that the species could be formally treated as subgenus *Stygoniphargus*, and that this species complex comprises seven subspecies. The species complex is diagnosed as follows.

Body. Slender, and may exceed 20 mm in length. Coxal plates as long as deep, or even slightly longer than deep ("flattened"). Epimeral plates (males only) I-II angular, III sometimes pointed. Mesosoma segment III with four thin setae along disto-posterior margins, rarely two or six. Urosoma segment I with a single dorso-postero lateral seta or weak spine, segment II two-three weak spines.

Antennae. Shorter than one half of the body length, aesthetascs shorter, one per flagellar article.

Gnathopods. Propods with inclined palm, propodus II (in males) as long as broad. Along outer margin of dactylus setae in groups of one to three.

Pereopods. Dactyls and their nail slender and narrow, with single spine at the base of nail.

Pereopods V-VII. With elongated, ovoid bases, posteriorly straight without distinct disto-posterior lobe.

Uropods. Uropod I of old males with endopodite as twice as long as exopodite. Uropod III in old males sometimes with elongated endopodite, distal and proximal article of expodpodite rod-shaped, elongated and of equal lengths.

Telson. With apical, lateral and sometimes dorsal spines.

Sexual dimorphism. Females have more pointed epimeral plates, stronger spines on telson, a broader telson cleft and non-differentiated rami of uropod I.

Already Karaman noted that some of the traits may deviate from the diagnostic combination. A part of deviations were described and named as subspecies (spine pattern of telson, elevated number of mesosomal setae, proportions on uropod III). Despite this, the diagnosis of the species complex is valid and reliable. By contrast, identification of species within the complex is highly unreliable. Our own observations indicate that many of diagnostic traits are variable, sex and age dependent. For detailed identification, readers should consult works of Karaman[8], Sket[7], and Karaman[9,10].

The cryptic nature of the *N. stygius* complex

Morphological crypsis means that species cannot be told apart on a basis of morphology alone, i.e. when intraspeciefic variation exceeds interspecific variation. The *N. stygius* species complex is such case. Populations differ from each other and this variation prompted S. Karaman to describe a series of subspecies[8]. Yet, discrimination of Karaman's subspecies is uncertain because intra- and inter-population variation exceeds inter-taxon variation. The taxonomy of this complex is impeded mainly by allometric and indeterminate growth[7], well studied also in many other niphargids[11]. Many diagnostic traits develop only in the largest males, but due to indeterminate growth it is often impossible to figure out at what stage of the development individual specimens are. Given that body length may evolve in relation to local conditions[12], it is difficult to estimate whether equally long individuals from different populations represent the same stage in ontogenetic development. We scrutinized series of populations for diagnostic traits of S. Karaman and additional putative diagnostic character, and summarize them in the Table S5. The diagnostic power of traits/characters decreases with the number of analyzed populations.

Table S5. Diagnostic characters proposed to discriminate subspecies diagnosed by S. Karaman. The problem related to diagnostic character is discussed at the bottom of the table.

Subspecies	Number of dorsal spines on telson1	Gnathopod II – number of setae groups on carpus*2	Gnathopods I-II number of supporting spines on propodus3	Uropod I inner ramus in males longer than outer ramus by factor4	Uropod III inner ramus elongated in males5	Mesosoma segments I-III – number of setae6
N. s. stygius	0	0	1	1.2-1.8	short	4-5
N. s. brachytelson	0	0-1	1	1.3-2.5	short	4
N. s. podpecanus	1-2	1	1	1.5-2.0	short	4
N. s. novomestanus	1-2 (3)	1	1	1.25-2.0*	short	4
N. s. likanus	1-2	1	1	1.5-2.0	short	4
N. s. karamani	2-6	1	1	1.5-2.5	short-long	4
N. s. kenki	2-7	0	1-3	1.5-2.0	short	4-6

[1]Number of spines depends on body size, comparisons between populations are not warranted. Moreover, intervals are broadly overlapping.

[2]This character relates to own observation and may be useful for discrimination of some species[13], although it depends on body size. However, polymorphism observed in herein studied taxa[14] diminishes the value of this trait in this complex.

[3]This character is rare and frequently occurs in Iranian *Niphargus*. However, we observed a morpho-cline among populations: virtually all individuals in eastern populations have multiple supporting spines whereas only few individuals in the western part of the range have multiplied spines.

[4]This character strongly depends on body size / age of an individual and is problematic *per se*. S Karaman. reported that the inner ramus of *N. s. stygius* and *N. s. novomestanus* is less elongated than in other taxa. However, we found strongly elongated inner rami in populations from both respective taxa in the catchment of Idrijca River and at the village Sela pri Straži.

[5]This character strongly depends on body size / age of an individual and is problematic *per se*. Karaman S. reported that it is strongly elongated in *N. s.karmani*, yet, we found populations with less elongated articles. Even if we did not have fully grown-up individuals at hand, this best illustrates the inoperability of this diagnostic trait.

[6]Setal ornamentation of mesosoma is a rather stable trait within the complex. Yet, there is some polymorphism on the between-population level in two subspecies, noted also in later works of S. Karman[15].

Newly described species

Niphargus goricae Delić, Trontelj & Fišer sp. n.

ZooBank lsid: A0A0651F-685D-45D4-9477-5E44408D4CF3

WoRMS lsid: 988195

Type locality: Water from the well by the house Fram 119, Fram, Maribor, Slovenia

Type series: *holotype* is a specimen with voucher number NA085, *paratype* is an intact adult male from the same sample. Samples are deposited in the Zoological collection of the Department of Biology, Biotechnical Faculty, Ljubljana.

Etymology: The name is derived from "Slovenske Gorice", a non-karstic region where the species is distributed.

Niphargus cvajcki Delić, Trontelj & Fišer sp. n.

ZooBank lsid: A6B6A0BC-20BC-4109-BAAA-72588484BC5A

WoRMS lsid: 988197

Type locality: Cave Šolnovo brezno, Prevole, Žužemberk, Slovenia

Type series: *holotype* is a specimen with voucher number NB915, *paratype* is a specimen with voucher number NB914. Samples are deposited in the Zoological collection of the Department of Biology, Biotechnical Faculty, Ljubljana.

Etymology: The name is derived from Slovenian autochthonous wine “Cviček”, in local dialect called “cvajčk”. The species is distributed in the area where this wine is produced.

Niphargus kapelanus Delić, Trontelj & Fišer sp. n.

ZooBank lsid: 10A49E2A-C93C-4114-A6EF-54374FE89AE1

WoRMS lsid: 988196

Type locality: Cave Špilja pod Mačkovom dragom, Bjelolasica, Ogulin, Croatia

Type series: *holotype* is a specimen with voucher number NB625. The sample is deposited in the Zoological collection of the Department of Biology, Biotechnical Faculty, Ljubljana.

Etymology: The name is derived from “Kapela”, a mountain on the border between the regions Lika and Gorski Kotar, at the bottom of which is type locality.

Niphargus iskae Delić, Trontelj & Fišer sp. n.

ZooBank lsid: 840AD88E-59E9-4969-ABBE-C4EFEB8D02D9

WoRMS lsid: 988194

Type locality: Spring on the foothill of Mačji rep, Škrabče, Nova vas, Slovenia

Type series: *holotype* is a specimen with voucher number NC087, *paratype* is an intact male from the same sample. Samples are deposited in the Zoological collection of the Department of Biology, Biotechnical Faculty, Ljubljana.

Etymology: The name is derived from river Iška, which cuts a canyon at northern boundary of Dinaric Karst in central Slovenia.

Remarks: The species was used in a study of photophobic response by Fišer et al. (2016), where it is referred to as *N. cf. podpecanus*.

Niphargus gottscheeanensis Delić, Trontelj & Fišer sp. n.

ZooBank lsid: 1EEEF2DC-8016-40CE-AB83-3E142905EAB2

WoRMS lsid: 988192

Type locality: Cave Željnske jame, Željne, Kočevje, Slovenia

Type series: *holotype* is a specimen with voucher number NB488, *paratype* is an intact male from the same sample. Sample are deposited in the Zoological collection of the Department of Biology, Biotechnical Faculty, Ljubljana.

Etymology: The name is derived from the old German name of Kočevje town. The town was first mentioned in late 14 century under the name *Gottschee*, and by that time controlled by counts of Ortenburg.

Niphargus malagorae Delić, Trontelj & Fišer sp. n.

ZooBank lsid: 333C1C2D-DF19-4B13-826D-C57782EF2864

WoRMS lsid: 988193

Type locality: Cave Mivčja jama, Gornje Lepovčje, Ribnica, Slovenia

Type series: *holotype* is a specimen with voucher number NB858, *paratype* is an intact male from the same sample. Samples are deposited in the Zoological collection of the Department of Biology, Biotechnical Faculty, Ljubljana.

Etymology: The name is derived from the geographic name of Mala Gora, a Dinaric massif where the type locality lies.

Niphargus chagankae Delić, Trontelj & Fišer sp. n.

ZooBank lsid: 4DF5BD7A-B343-4354-AC65-F7FFFA829123

WoRMS lsid: 988191

Type locality: Cave Čaganka, Poljanska gora, Črnomelj, Slovenia

Type series: *holotype* is a specimen with voucher number NB792, *paratype* is an intact male from the same sample. Samples are deposited in the Zoological collection of the Department of Biology, Biotechnical Faculty, Ljubljana.

Etymology: The name is derived from the name of the type locality, cave Čaganka.

Niphargus kordunensis Delić, Trontelj & Fišer sp. n.

ZooBank lsid: 2A2EBA04-D1C8-4024-A1B0-D9BBE0E93CFD

WoRMS lsid: 988190

Type locality: Matešička špilja, Matešići, Slunj, Croatia

Type series: *holotype* is a specimen with voucher number NB623, *paratype* is a specimen with voucher number NC089. Samples are deposited in the Zoological collection of the Department of Biology, Biotechnical Faculty, Ljubljana.

Etymology: The name is derived from the geographical name Kordun, a region in central Croatia, where the type locality is situated.

References

1. Lanfear, R., Calcott, B., Ho, S. Y. W. & Guindon, S. PartitionFinder: Combined selection of partitioning schemes and substitution models for phylogenetic analyses. *Mol. Biol. Evol.* **29,** 1695–1701 (2012).
2. Verovnik, R., Sket, B. & Trontelj, P. The colonization of Europe by the freshwater crustacean Asellus aquaticus (Crustacea: Isopoda) proceeded from ancient refugia and was directed by habitat connectivity. *Mol. Ecol.* **14,** 4355–4369 (2005).
3. Colgan, D. *et al.* Histone H3 and U2 snRNA DNA sequences and arthropod molecular evolution. *Aust. J. Zool.* **46,** 419–437 (1998).
4. Folmer, O., Black, M., Hoeh, W., Lutz, R. & Vrijenhoek, R. DNA primers for amplification of mitochondrial cytochrome c oxidase subunit I from diverse metazoan invertebrates. *Mol. Mar. Biol. Biotechnol.* **3,** 294–299 (1994).
5. Fišer, Ž., Altermatt, F., Zakšek, V., Knapič, T. & Fišer, C. Morphologically cryptic Amphipod species sre ‘ecological clones’ at regional but not at local scale: a case study of four Niphargus species. *PLoS One* **10,** e0134384 (2015).
6. Flot, J.-F., Wörheide, G. & Dattagupta, S. Unsuspected diversity of Niphargus amphipods in the chemoautotrophic cave ecosystem of Frasassi, central Italy. *BMC Evol. Biol.* **10,** 1–18 (2010).
7. Sket, B. Niphargus stygius (Schiödte) (Amphipoda, Gammaridae)- die Neubeschreibung des Generotypus, Variabilität, Verbreitung und Biologie der Art, I. *Biološki Vestn.* **22,** 91–103 (1974).
8. Karaman, S. Podrod Stygoniphargus u Sloveniji i Hrvatskoj. *Prirodosl. istraživanja* **25,** 5–38 (1952).
9. Karaman, G. S. Two poorly known species of the subterranean family Niphargidae (Gammaridea) from Slovenia and Croatia (Contribution to the Knowledge of the Amphipoda 276). *Nat. Montenegrina* **13,** 281–308 (2014).
10. Karaman, G. S. Niphargus cerjanensis, sp. n. and N. karamani Schell. 1935 from the subterranean waters of Western Balkan (Contribution to the knowledge of the Amphipoda 277). *Agric. For.* **60,** 223–256 (2014).
11. Fišer, C., Bininda-Emonds, O. R. P., Blejec, A. & Sket, B. Can heterochrony help explain the high morphological diversity within the genus Niphargus (Crustacea: Amphipoda)? *Org. Divers. Evol.* **8,** 146–162 (2008).
12. Delić, T., Trontelj, P., Zakšek, V. & Fišer, C. Biotic and abiotic determinants of appendage length evolution in a cave amphipod. *J. Zool.* **299,** 42–50 (2016).
13. Fišer, C., Sket, B. & Stoch, F. Distribution of four narrowly endemic Niphargus species (Crustacea: Amphipoda) in the western Dinaric region with description of a new species. *Zool. Anz.* **245,** 77–94 (2006).
14. Fišer, C. & Zagmajster, M. Cryptic species from cryptic space: the case of Niphargus fongi sp. n. (Amphipoda, Niphargidae). *Crustaceana* **82,** 593–614 (2009).
15. Karaman, S. L. Die Niphargiden des slovenischen Karstes, Istriens sowie des benachb. Italiens. *Acta Musei Maced. Sci. Nat.* **3,** (1954).
